# Identification of a molecular marker associated with lignotuber in *Eucalyptus ssp*

**DOI:** 10.1038/s41598-020-60308-8

**Published:** 2020-02-27

**Authors:** Tânia M. Bortoloto, Maria C. P. Fuchs-Ferraz, Karine Kettener, Lígia Martins Rubio, Esteban R. González, Izabel C. G. de Souza, Shinitiro Oda, Bruno C. Rossini, Celso L. Marino

**Affiliations:** 1 Departamento de Genética, Instituto de Biociências, UNESP - Univ Estadual Paulista, R. Prof. Dr. Antônio Celso Wagner Zanin s/n, Botucatu, SP CEP 18618-689 Brazil; 2 Suzano Papel e Celulose SA, Av. Dr. José Lembo 1010, Itapetininga, SP CEP 18207-780 Brazil; 3 Instituto de Biotecnologia (IBTEC), UNESP - Univ Estadual Paulista, Alameda das Tecomarias s/n, Botucatu, SP CEP 18607-440 Brazil

**Keywords:** Agricultural genetics, Plant genetics

## Abstract

About 95% of *Eucalyptus* species present an organ known as a lignotuber, a basal woody swelling that holds a large number of dormant buds in a protected position along with carbohydrates and other nutrients. The importance of this trait in *Eucalyptus* species relates to its regenerative capacity, particularly in the context of coppicing practices and survival in regions of high abiotic stress, especially fire. In this study, we identified and characterized a genomic region associated with the lignotuber trait in commercially important *Eucalyptus* species by developing a polymorphic marker that co-segregates with lignotuber presence. The marker was then converted into a SCAR (Sequence Characterized Amplified Region) marker, validated in four other *Eucalyptus* species and hybrids and analyzed *in silico*. Our investigation presents a marker (*ELig*) that is effective in identifying individuals with lignotuber. *In silico* and Southern blot analyses show that the marker is present in a single copy region and is related to auxilin/cyclin-G associated kinase, containing a DnaJ domain. The *ELig* marker is an important tool that can be used to manage crosses in *Eucalyptus* breeding programs and inform studies involving lignotuber development and genetics.

## Introduction

*Eucalyptus* is a widely planted tree genus due to its ability to adapt, grow, and produce quality wood that can be used for multiple end purposes^1,2^. In Brazil, *Eucalyptus* plantations are extremely important as they comprise more than 71% of the total forest plantation area (5.56 million ha), making the country a leader in the forestry sector^3^. In this context, researchers and foresters seek to implement tree improvement programs, advance silvicultural practices to increase productivity, introduce desirable traits, and reduce the environmental impacts of eucalypt plantations^4–6^. A common practice in *Eucalyptus* management is coppicing, which offers an initial sprout growth rate greater than planting seedlings and is enhanced by the presence of an organ known as a lignotuber^7–9^.

Natural conditions in Australia led to the evolution of the *Eucalyptus* genus which is strongly associated with the occurrence of fire. Trees have been both survivors and promoters of fire over millions of years, leading to the development of a series of regeneration strategies including lignotubers^10^. Lignotubers are basal, woody swellings that can form rapidly during the seedling stage^11–13^. They hold a large number of dormant buds in a protected position, along with carbohydrates and nutrients necessary for bud development^11,12,14^. Under normal conditions, the buds remain dormant, but they can be activated by stress factors, such as defoliation^14^, fire and coppicing^9,15^, and nutrient and water deficiencies^13,16^, underscoring the importance of the lignotuber for tree survival. These organs are present in 95% of all *Eucalyptus* species, including *E. urophylla*, *E. brassiana*, and *E. saligna*; however, some commercial species, such as *E. grandis*, *E. regnans*, *E. delegatensis*, *E. pilularis*, and *E. nitens*, do not possess lignotubers^17–20^.

Given the regenerative potential of lignotubers, there are clear advantages to introducing this trait into commercial eucalypt species through breeding programs. In this study, we identify and characterize a genomic region associated with the lignotuber trait in *Eucalyptus* in a 3:1 genetic control ratio, indicating a dominant genetic effect on lignotuber formation. Our investigation offers a better understanding of the copy number of the associated region in the *E. grandis* and *E. urophylla* genome and the efficiency of the *ELig* marker to identify individuals with lignotuber.

## Materials and Methods

### Plant material

In order to select individuals who effectively develop the organ or not, we used a cross between *Eucalyptus grandis* × *E. urophylla*, which generated a F1 hybrid called “*Eucalyptus* urograndis” from Suzano Papel and Celulose SA company. The F1 offspring were crossed generating F2 seeds that were sown in a greenhouse. Over a period of 12 months, the resulting 111 individuals were submitted to conditions of hydric and nutrient stress in order to induce lignotuber development; seedlings were constantly monitored to identify the presence or absence of lignotuber. The proportion of individuals showing lignotuber presence was tested using a chi-squared (${\chi }^{2}$) test. For validation tests, we used F2 hybrid material (“*E. urograndis*”) with a different genetic background from another Brazilian pulp and paper company that maintains a separate breeding program. Finally, four other species of *Eucalyptus* (*E. grandis, E. urophylla, E. saligna* and *E. brassiana*), that constitutively exhibit the presence/absence of lignotuber, were used in order to confirm our results. The use of the plant material was authorized by Fibria Celulose SA and Suzano Papel and Celulose SA companies, and the study did not involve endangered or protected species.

### Genomic DNA extraction

Genomic DNA was isolated from fresh leaves using the CTAB method^21^ with modifications. Approximately 150 mg of fresh leaf material was ground in liquid nitrogen to a fine powder, homogenized in 700 µl of CTAB extraction buffer (2% CTAB, 2% PVP, 100 mM Tris-HCl, pH = 8.0, 25 mM EDTA, 2 M NaCl, 0.2% 2-mercaptoethanol) and incubated at 65 °C for 45 min. A total of 600 µl of chloroform-isoamyl alcohol (CIA 24:1) was added, then the mixture was vortexed and centrifuged for 5 min at 10,000 g. The supernatant was transferred to a new tube, 400 µl of chilled isopropyl alcohol was added and then incubated overnight at −20 °C. The samples were subsequently centrifuged for 5 min at 10,000 g, the supernatant discarded, and the pellet washed with 95% ethanol. The DNA was resuspended in 80 µl TE buffer (10 mM Tris, 1 mM EDTA, pH = 8.0) containing 10 ng/µl of RNAse. Total DNA concentration was quantified by spectrophotometer (NanoDrop*™* 1000, Thermo Scientific) and visualized on 1% agarose gel in 1X TBE (Tris-borate-EDTA) stained with ethidium bromide.

### Molecular marker development and validation

For molecular marker identification, we used the Bulked Segregant Analysis (BSA) technique^22^. In order to identify genetic markers, we created two bulks of the studied hybrids (*E. urograndis*, with equal amounts of genomic DNA); one bulk included plants with lignotuber and another included plants without lignotuber from our greenhouse experiment (Bulks 1 and 2, respectively). Three DNA pools (Table 1) were also used to test if the detected molecular polymorphism is transferable between different germplasm provenances. The DNA pools consisted of genetic materials from other independent F2 *E. grandi*s x *E. urophylla* crosses and also from other species (*E. saligna)*.

**Table 1:** Description of the bulks and DNA pools analyzed in this study from two different of pulp and paper companies and their respective number of individuals.

ID sample	Species	Presence or absence of lignotuber	Number of plants
Bulk 1	*E. urograndis* from Suzano Papel and Celulose SA company (F2)	Presence	5
Bulk 2	*E. urograndis* from Suzano Papel and Celulose SA company (F2)	Absence	5
DNA pool 1	*E. urograndis* self-pollination from Fibria Celulose SA company (F2)	Absence	10
DNA pool 2	*E. urograndis* self-pollination from Fibria Celulose SA company (F2)	Presence	9
DNA pool 3	*E. saligna*	Presence	5

Initially, the two DNA bulks (Bulks 1 and 2) were screened for polymorphic markers using 147 random primers (kits AD, W, M, K, R, Y, Q, and X; Operon Technologies, Inc.). Only primer OP-X07 showed one band that co-segregates with lignotuber trait. The reaction consisted of 9 ng DNA, 1.30 μl 10X PCR Buffer (Invitrogen), 1 mM MgCl_2_, 1.04 μl BSA at 10 mg/mL (Bovine Serine Albumin; Invitrogen), 1 mM dNTP mix, 0.3 μM of primers, 1 U of Taq DNA polymerase (Invitrogen), and water to a final volume of 10 μl. PCR was performed under the following conditions: initial denaturation at 92 °C for 3 min; 40 cycles of 92 °C for 1 min; 35 °C for 1 min; 72 °C for 2 min; and a final extension at 72 °C for 5 min. Amplified products were visualized on 1% agarose gel in 1X TBE stained with ethidium bromide. Subsequently, a validation step was performed for the DNA pools from distinct provenances (DNA pools 1 and 2; Table 1) and also for four other *Eucalyptus* species (Table 2). The DNA pools were used as an initial confirmation screening and afterwards the marker were tested for each individual of the different species (Table 2). This approach enabled us to include as many samples as possible in one reaction, where the results consistently showed presence/absence of the marker. Secondly, we selected a polymorphic band that co-segregates with lignotuber presence and converted it into a SCAR marker, eliminating RAPD (Random Amplified Polymorphic DNA) non-reproducibility.

**Table 2:** Efficacy test of *ELig* marker in four *Eucalyptus* species. +: species with lignotuber development, −: species without lignotuber development.

Species	Lignotuber presence	Number of individuals genotyped	Number of individuals with *ELig* marker	Marker efficiency
*E. urophylla*	+	77	1	98.7%
*E. grandis*	−	69	61	88.4%
*E. saligna*	+	10	1	90%
*E. brassiana*	+	8	0	100%

The RAPD bands were extracted from the agarose gel and purified with Illustra GFX PCR DNA kits (GE Healthcare). Purified fragments were cloned into the pGEM-T Easy Vector (Promega) and inserted into UltraMAX DH5α-FT competent *Escherichia coli* cells (Life Technologies), following the manufacturer’s protocol. Plasmid DNA was isolated using a miniprep assay^23^ and the DNA was resuspended in ultrapure water. Plasmid DNA was quantified by spectrophotometer.

Purified plasmid DNA with RAPD fragments were bi-directionally sequenced on ABI3130xl Genetic Analyzer (Applied Biosystems) with BigDye Terminator Cycle Sequencing kit v. 3.1 (Applied Biosystems) according to the manufacturer’s instructions.

Based on the sequence information of the cloned RAPD fragments and current literature^24–26^, primers were designed to characterize the SCAR, herein denominated *ELig*. The *Elig* sequence consists of an 857 bp region amplified with the forward primer *ELIg-F* 5′-GAGCGAGGCTAATTAGTCC-3′, and reverse primer *ELig-R* 5′-CTTACCAGAGAGCGAGGC-3′ (GenBank accession number MK680035). We validated the marker against 164 individuals from four *Eucalyptus* species (*E. grandis* - 69 plants; *E. urophylla* - 77 plants; *E. saligna* - 15 plants; and *E. brassiana* − 8 plants) to determine marker transferability and efficacy. The SCAR reaction consisted of 25 ng DNA, 1 μl 10X PCR Buffer (Invitrogen), 1.5 mM MgCl_2_, 1 mM dNTP mix, 0.5 μM of each primer, 0.5 U of *Taq* DNA polymerase (Invitrogen), and water to a final volume of 10 μl. PCR was performed under the following conditions: initial denaturation at 94 °C for 5 min, followed by 35 cycles of 94 °C for 30 s, 64 °C for 1 min, 72 °C for 1 min 35 s, and a final extension at 72 °C for 7 min. Amplified products were visualized after electrophoresis on 1% agarose gel in 1X TBE stained with ethidium bromide.

### Southern blot

Genomic DNA (20 μg) from *E. grandis* and *E. urophylla* was digested with restriction enzymes *EcoR*I, *Nde*I, and *Xba*I, electrophoresed on a 0.7% agarose gel and transferred to Hybond-N + nylon membrane (Amersham). For probe construction, the *ELig* region was amplified and the fragment purified using PureLink Quick Gel Extraction Kit (Invitrogen). The fragments were labeled using AlkPhos Direct Labeling and Detection Systems Kit (GE Healthcare, UK). Hybridization and detection were performed as per the manufacturer’s instructions.

### *In silico* analysis

The *ELig* sequence was compared with the eucalypt genome from the Phytozome v. 12.1.6 database (*Eucalyptus grandis* v2.0 - https://phytozome.jgi.doe.gov/pz/portal.html) using the tool BLAST^27^. Previous studies based on DArT markers reported an average physical distance per centiMorgan of 633 kb with a range of 100 kb to 2.4Mb^28^. Thus, we searched against previous studies of linkage and physical maps for microsatellite^29^, DArT^30^ and SNPs^31^ in ~1 cM (633 kb) to find markers related to the *Elig* marker. For this, we used BLAST to search for the closest markers described in physical maps next to *ELig*. The final integrative map of these studies and the *ELig* sequence was developed with genoPlotR^32^. In addition, to search for possible genes related to responses to abiotic stress^33^, we expanded the search to a range of ~2 cM (1.3 Mb) from the *Elig* sequence, conducting a Gene Ontology analysis using Blast2GO software.

## Results

Two phenotypic classes (presence and absence of lignotuber) were identified in the F2 progeny of 111 individuals. Three individuals did not survive. Seventy-four showed the presence of a lignotuber while thirty-four did not. The Chi-square (χ2 = 2.42; p-value >0.1) test indicates that the progeny segregate in a ratio of 3:1 (with lignotuber: without lignotuber), suggesting Mendelian segregation of the trait (Supplementary Figure 1).

Of all the random primers tested, only primer OP-X07 revealed polymorphism between the plants with and without lignotuber, co-segregating with the phenotype. A fragment with approximately 850 bp was found only in individuals from the bulk and DNA pools without lignotuber. The random primer site was fully complementary with the 5′ end of the marker sequence; however, in the 3′ end, only the first 6 nucleotides of the primer were complementary. This indicates that non-amplification of the primer in individuals with the lignotuber was due to non-pairing of the four bases of the primer in the 3′ end of the region.

The *ELig* marker, as well as amplification with the OP-X07 random primer, showed bands with the same size (850 bp) in Bulk 2 and DNA pool 1 (data not shown). When tested in individuals from *E. grandis* and *E. urophylla*, the marker only showed amplification in individuals without lignotuber (Supplementary Figure 2). Marker efficiency was 88.4% in *E. grandis* and up to 100% in species with lignotuber. Meanwhile, for *E. urophylla* the efficacy of the marker was 98.7% and for *E. saligna* it was 90% (Table 2). Thus, the mean efficacy of the marker in identifying the presence or absence of the lignotuber was 93.9%. Both genomes of *E. grandis* and *E. urophylla* in the Southern blot analysis indicated the presence of only one copy of the marker selected (Supplementary Figure 3).

The search against Phytozome revealed a single region with score 1,256.4, e-value 0.0 at chromosome 04 in the *E. grandis* genome (other results produced non-significant alignments). The identification of this significant and unique region is consistent with the results obtained by Southern blot. The BLAST alignment of the *ELig* sequence resulted in high similarity with the 3′ UTR region of the Eugr.D00271 gene, that encodes two other variant transcripts, consisting of seven exons. This gene consists of 3091 bp and is related to auxilin/cyclin-G-associated kinase (PTHR23172) and auxilin-like protein and related proteins containing DnaJ domain (KOG0431), which are closely linked to abiotic stress responses.

**Figure 1:**
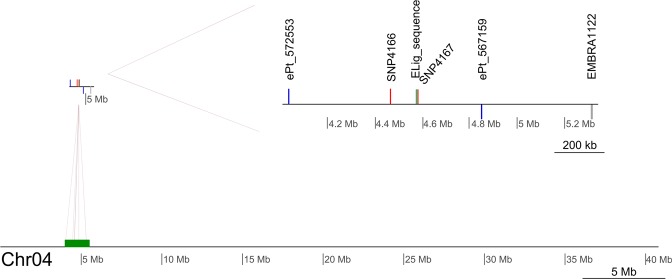
Integrative map of SNP, microsatellite and DArT markers within the *ELig* sequence.

An integrative map with other physically mapped markers resulted in two SNPs, two DArTs and one microsatellite marker surrounding the *ELig* marker (Fig. 1). Furthermore, the GO analysis revealed a large number of genes that are members of the cytochrome p450 family, ATP-binding cassette transporters (ABC transporters) and purine permeases (see Supplemental Table 1).

## Discussion

The persistence of species despite a range of environmental challenges has been studied in eucalypts as examples of species evolution due to clear responses to fire and drought stress^10^. The analysis of genome-wide DArT markers in terms of the reproductive strategy of eucalypt species through seeding (obligate-seeders) or regeneration (lignotuber-resprouter) demonstrate that there are differences between them, with the lignotuber state not considered phenotypic plasticity in response to environmental conditions, but rather genetically controlled^34^. On the other hand, when working with hybrids, the presence of lignotuber in F2 crosses is induced by environmental conditions. Unfortunately, similar results to this study for the ratio of lignotuber presence/absence (3:1) were not obtained for *Corymbia*; a F2 cross from *Corymbia torelliana* × *Corymbia citriodora* was assessed for QTLs related to the presence/absence of lignotuber, where the QTL identified all homospecific parental alleles from *C. citriodora variegata* as having lignotuber and those from *C. torelliana* parental alleles as without^35^. These results could be related to cis- and trans-regulatory divergence between parental species, with the occurrence of association of chromatin modifications as demonstrated for *Arabidopsis*. This results in a complex interaction where alleles from one parent up- or down-regulate the gene expression in the other, while also showing the dominance of one species in gene expression^36^.

The presence of a marker suitable to identify a region associated with lignotuber development in *Eucalyptus*, as well as the physical linkage with other markers, enhances our understanding of the possible genetic mechanisms involved in this organ. Furthermore, the fact that the *Elig* sequence is present in a gene involved in responses to abiotic stress, as with the other physically proximal genes, could help to elucidate the nature of regeneration in the face of environmental hardship and selection of individuals for breeding programs.

Association studies based on linkage disequilibrium in populations are used to identify related alleles with a given phenotype^37^. Our results indicate that the association between the *ELig* marker and the lignotuber phenotype occurs in a single copy in the genome. Single copy number genes are found in low numbers in *Eucalyptus* (~18.9% of genes) and other plant genomes^38^.

Although random primers offer numerous advantages, sensitivity to changes in the reaction conditions makes the results difficult to reproduce. Another disadvantage is the possibility of errors in data interpretation due to the co-migration of fragments with equal or very similar sizes or bands in the same position on two different individuals originating from different loci^39^. To overcome these issues, the marker that is linked to the absence of lignotuber was converted into a SCAR marker. SCAR markers are less sensitive, more informative (as a codominant marker), and generate more straightforward results due to the detection of only a single locus^24,26,40^.

One issue that could affect marker efficiency is the purity of eucalypt species in Brazil. The natural occurrence of interspecific eucalypt crosses has resulted in the formation of hybrids in the Brazilian germplasm. It is common for species that would be geographically isolated in Australia to be planted in close proximity in Brazil, thus increasing the possibility of producing hybrids. For example, when *E. urophylla* was introduced into Brazil in Rio Claro in 1919^41^ it was planted together with *E. tereticornis* and *E. saligna*. It is very likely that the populations developed from these *E. urophylla* seeds are hybrids of these three species. This situation is also likely for subsequent generations of *E. grandis* since both *E. urophylla* and *E. grandis* flower ten months a year^42^. While there is no way to measure the level of hybridization of these species in the Brazilian germplasm and in pulp and paper company plantations, hybridization could be the main reason for the failure to detect the marker in some individuals.

**Figure 2:**
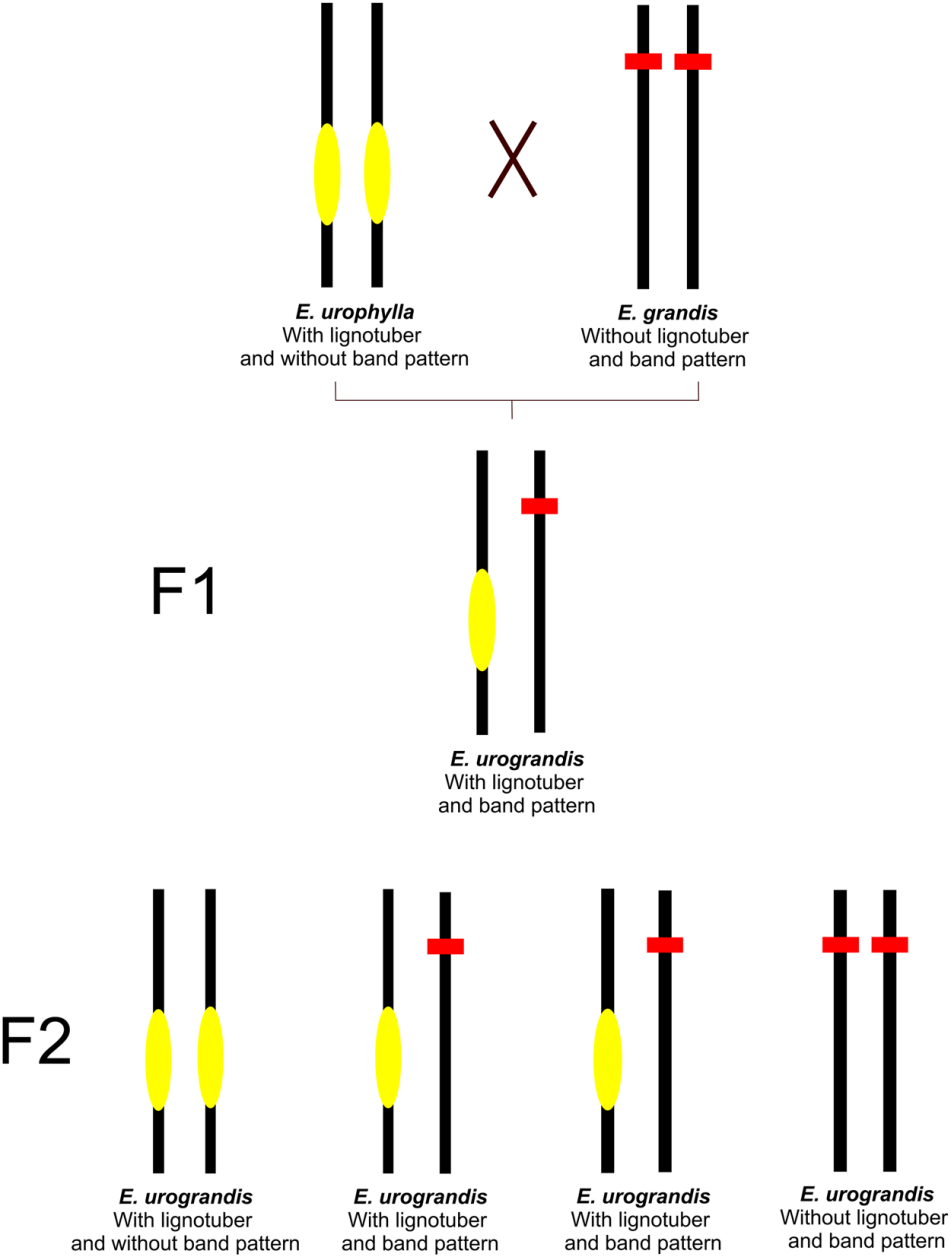
Recombination of *Elig* marker and the genetic effect of lignotuber in crosses of *E. grandi*s × *E. urophylla*. In yellow, the probable QTL related to lignotuber organ development and in red the *Elig* marker. Black bars represent chromosomes. In the F2 cross, it is expected that two thirds of the individuals show the *Elig* marker.

Although the *ELig* marker is highly effective and transferable to other populations, it is clear that a low frequency of individuals carrying the marker also show lignotuber presence, while some individuals without the marker may not present the trait. In addition to the genetic issue of species hybridization, this could be related to recombination between the marker and target regions responsible for lignotuber development. In fact, when analyzing the F2 hybrids, we must point out that recombination could lead to misidentification, where the presence or absence of lignotuber would be incorrectly estimated (Fig. 2). This result confirms that the *Elig* sequence is closely linked to another gene that controls lignotuber development, as suggested by the study with *Corymbia*^35^.

In order to clarify this issue, we analyzed the marker *in silico* and identified that the region of the *Elig* sequence (Eugr.D00271) has a DnaJ domain. Although the *Elig* sequence is not directly responsible for the presence of lignotuber, its function could be related to a cluster of genes involved in stress responses. Structurally, DnaJ protein consists of a conserved N-terminal domain called “J domain”, a region rich in glycine (glycine-rich - ‘G’ domain’), a central domain containing four repeats of CXXCXGXG motif (CRR domain) and a C- terminal domain^43^. The J domain is highly conserved and present in several prokaryotic and eukaryotic proteins of divergent organism groups^44^. Proteins of the DnaJ family, in general, function as chaperones and are involved in various processes in intracellular machinery, enforcing and maintaining the native structure of other proteins^45^. DnaJ is a member of the Hsp40 (40-kDa heat-shock protein) family that regulates the activity of Hsp70s (70-kDa heat-shock proteins)^46^, where Hsp70s are needed to import proteins into the eukaryotic cell organelle, such as the chloroplast, endoplasmic reticulum, and lysosome. Hsp70 participates in folding, facilitating membrane translocation of the peptide through the membrane pore and allowing its importation into the organelle^47^. Hsp proteins can accumulate in response to abiotic stress and there is a positive correlation between the amount of Hsp proteins and the degree of thermotolerance^48–50^. The association of these proteins with responses to abiotic stress reinforces the presence of lignotuber as an adaptation to xerophytic and/or fire-prone environments^19,20,51,9^ and its formation can be stimulated by stress events^19,52^.

Although the evolutionary history of *Corymbia* and *Eucalytpus* are closely related^53^, the BLAST analysis revealed different control regions for the lignotuber trait in each genera when we considered the QTL linkage groups found for lignotuber in *Corymbia*^35^; both markers EMCRC40 and EMBRA40 (located in chromosome 11 in *Corymbia*) are present in chromosome 10 of the *E. grandis* genome. Although control over lignotuber occurs in different regions for the two genera, the *Corymbia* study suggests that lignotuber development may be multigenic with dominant gene effects, supporting the results presented herein. The GO analysis in the *E. grandis* genome indicated that several genes near the *Elig* marker region are members of the cytochrome p450 family, which are very important genes for plant growth and response to biotic and abiotic stresses^54^. Other genes are well represented in the chromosome 4 region of the *Elig* sequence, such as ERD4 (early-response to dehydration stress protein) which is involved in responses to abiotic stress^55^, including enhanced tolerance of salt and drought in *Arabidopsis*^56^. Further, several genes related to purine permease, which are involved in growth and development control in rice, were found in this region^57^, as well as starch synthase (Eucgr.D00323), genes from the photosystem, such as light-harvesting complex II chlorophyll a/b binding protein 1 (from Eucgr.D00319 to Eucgr.D00322), and several transcription factors that contribute to energy reserves and plant development. Although we did not evaluate gene expression, the presence of a high number of stress related genes indicates possible target genes that will serve as the basis for future studies.

## Conclusions

The characterization of a marker associated with lignotuber development is highly significant for *Eucalyptus* as individuals that have this organ can regenerate quickly after coppicing or fire. It is thus a potential marker for selection in breeding programs. In addition, the identification of closely related markers are helpful in assessing this characteristic, while the GO analysis revealed that several genes linked to abiotic stress responses are located near to the *Elig* sequence region. As such, it was possible to characterize the genes in the marker region, serving as foundational support for future studies in gene expression and physiology of the lignotuber organ.

## Supplementary information

Supplementary material.

## References

[1] Grattapaglia D, Kirst M (2008). *Eucalyptus* applied genomics: from gene sequences to breeding tools. New Phytol..

[2] Myburg A (2008). Forest and fibre genomics: biotechnology tools for applied tree improvement. South For. J. For. Sci..

[3] IBÁ - Brazilian Tree Industry. Report of the Brazilian Tree Industry - base year 2014 (2015).

[4] Golle DP, Reiniger LRS, Curti AR, Bevilacqua CB (2009). Forestry improvement: emphasis on biotechnology application. Cienc. Rural, Santa Maria..

[5] Rockwood, D.L. History and Status of *Eucalyptus* Improvement in Florida. *Int. J. For. Res*. Article ID 607879 (2012).

[6] Gonçalves JLM (2013). Integrating genetic and silvicultural strategies to minimize abiotic and biotic constraints in Brazilian eucalypt plantations. Forest Ecol. Manag..

[7] Stape, J. L. *et al*. Manejo de brotação de *Eucalyptus spp*: resultados técnico-operacionais. Circular Técnica IPEF. 183 (1993).

[8] Reis GG, Reis MDGF (1997). Fisiologia da brotação de eucalipto com ênfase nas suas relações hídricas. Série técnica IPEF..

[9] Whittock SP (2003). Genetic control of coppice and lignotuber development in Eucalyptus globulus. Aust. J. Bot..

[10] Hill RS (2016). Evolution of the eucalypts - an interpretation of the macrofossil record. Aust. J. Bot..

[11] Carrodus BB, Blake TJ (1970). Studies on the lignotubers of Eucalyptus obliqua L’Heri. New Phytol..

[12] James S (1984). Lignotubers and burls—their structure, function and ecological significance in Mediterranean ecosystems. The Botan. Rev..

[13] Walters JR, House AP, Doley D (2005). Water and nutrient availabilities do not affect lignotuber growth and sprouting ability of three eucalypt species of south-eastern Queensland. Aust. J. Bot..

[14] Graham AW, Wallwork MA, Sedgley M (1998). Lignotuber bud development in Eucalyptus cinerea (F. Muell. ex Benth). Int. J. Plant Sci..

[15] Noble JC (2001). Lignotubers and meristem dependence in mallee (*Eucalyptus spp*.) coppicing after fire. Aust. J. Bot..

[16] Neave LA, Florence RG (1998). Moisture stress stimulates the subsequent growth of lignotuberous *Eucalyptus maculata* seedlings. Austral. For..

[17] Jacobs, M. R. Growth habits of the eucalypts. Forestry and Timber Bureau, Camberra (1955).

[18] Eldridge, K., Davidson, C., Harwood, C. & Van Wyk, G. *Eucalyptus* domestication and breeding. Oxford University Press, New York (1994).

[19] Brooker, I. Botany of the eucalypts. In: Coppen JJW, editor. *Eucalyptus*: the genus *Eucalyptus*. Taylor & Francis, London (2002).

[20] Rejmánek, M. & Richardson, D. M. Eucalypts. In: Simberloff D, Rejmánek M, editors. Encyclopedia of biological invasions. University of California Press, Berkeley (2011).

[21] Doyle JJ, Doyle JL (1987). Isolation of plant DNA from fresh tissue. Focus..

[22] Michelmore RW, Paran I, Kesseli RV (1991). Identification of markers linked to disease-resistance genes by bulked segregant analysis: A rapid method to detect markers in specific genomic regions by using segreganting populations. Proc. Natl. Acad. Sci..

[23] Sambrook, J. & Russel, D. W. Preparation of Plasmid DNA by Alkaline Lysis with SDS: Minipreparation. CSH Protoc. (1)pii:pdb.prot4084 (2006). 10.1101/pdb.prot4084 22485489

[24] Barret P, Delourme R, Foisset N, Renard M (1998). Development of a SCAR (sequence characterized amplified region) marker for molecular tagging of the dwarf BREIZH (Bzh) gene in *Brassica napus* L. Theor. Appl. Genet..

[25] Hernández P, Martín A, Dorado G (1999). Development of SCARs by direct sequencing of RAPD products: a practical tool for the introgression and marker-assisted selection of wheat. Mol. Breeding..

[26] Negi MS, Devic M, Delseny M, Lakshmikumaran M (2000). Identification of AFLP fragments linked to seed coat colour in *Brassica juncea* and conversion to a SCAR marker for rapid selection. Theor. Appl. Genet..

[27] Altschul SF (1997). Gapped BLAST and PSI-BLAST: a new generation of protein database search programs. Nucleic Acids Res..

[28] Kullan ARK (2012). High-density genetic linkage maps with over 2,400 sequence-anchored DArT markers for genetic dissection in an F2 pseudo-backcross of Eucalyptus grandis x E. urophylla. Tree Genet. Genomes..

[29] Grattapaglia D, Mamani E, Silva‐Junior OB, Faria DA (2015). A novel genome‐wide microsatellite resource for species of *Eucalyptus* with linkage‐to‐physical correspondence on the reference genome sequence. Mol. Ecol. Resour..

[30] Petroli CD (2012). Genomic characterization of DArT markers based on high-density linkage analysis and physical mapping to the eucalyptus genome. PLoS One.

[31] Bartholomé JI (2015). High-resolution genetic maps of Eucalyptus improve Eucalyptus grandis genome assembly. New Phytol..

[32] Guy L, Kultima JR, Andersson SGE (2010). genoPlotR: comparative gene and genome visualization in R. Bioinformatics..

[33] Conesa A (2005). Blast2GO: a universal tool for annotation, visualization and analysis in functional genomics research. Bioinformatics..

[34] Gosper CR (2019). Phylogenomics shows lignotuber state is taxonomically informative in closely related eucalypts. Mol Phylogenet Evol..

[35] Shepherd M (2008). Mapping species differences for adventitious rooting in a *Corymbia torelliana* × *Corymbia citriodora* subspecies variegata hybrid. Tree Genet Genomes..

[36] Shi X (2012). Cis‐ and trans‐regulatory divergence between progenitor species determines gene‐expression novelty in *Arabidopsis* allopolyploids. Nat Commun..

[37] Andersen JR, Lübberstedt T (2003). Functional markers in plants. Trends Plant Sci..

[38] Han F (2014). Identification, characterization, and utilization of single copy genes in 29 angiosperm genomes. BMC Genomics..

[39] Paran I, Michelmore RW (1993). Development of reliable PCR-based markers linked to downy mildew resistance genes in lettuce. Theor. Appl. Genet..

[40] Agarwal M, Shrivastava N, Padh H (2008). Advances in molecular marker techniques and their applications in plant science. Plant Cell Rep..

[41] Andrade, E. N. O eucalipto. Companhia Paulista de Estradas de Ferro, Jundiaí, SP (1961).

[42] Pryor LD (1971). Aspectos da cultura do eucalipto no Brasil. IPEF..

[43] Finn RD (2008). The Pfam protein families database. Nucleic Acids Res..

[44] Cheetham ME, Caplan AJ (1998). Structure, function and evolution of DnaJ: conservation and adaptation of chaperone function. Cell Stress Chaperon..

[45] Miernyk JA (2001). The J-domain proteins of Arabidopsis thaliana: an unexpectedly large and diverse family of chaperones. Cell Stress Chaperon..

[46] Walsh P, Bursać D, Law YC, Cyr D, Lithgow T (2004). The J‐protein family: modulating protein assembly, disassembly and translocation. EMBO reports.

[47] Mayer MP, Bukau B (2005). Hsp70 chaperones: cellular functions and molecular mechanism. Cell Mol. Life Sci..

[48] Park J, Seo YS (2015). Heat shock proteins: a review of the molecular chaperones for plant immunity. Plant Pathol..

[49] Jacob P, Hirt H, Bendahmane A (2017). The heat shock protein/chaperone network and multiple stress resistance. Plant Biotech..

[50] Lin MY (2014). A positive feedback loop between HEAT SHOCK PROTEIN101 and HEAT STRESS-ASSOCIATED 32-KD PROTEIN modulates long-term acquired thermotolerance illustrating diverse heat stress responses in rice varieties. Plant Physiol..

[51] Molinas ML, Verdaguer D (1993). Lignotuber ontogeny in the cork-oak (*Quercus suber*, Fagaceae). II. Germination and young seedling. Am. J. Bot..

[52] Del Tredici, P. Lignotuber development in *Ginkgo biloba*. In: Hori, T. *et al*. editors. The World of Ginkgo. Springer Verlag, Tokyo (1997).

[53] Thornhill AH (2015). Interpreting the modern distribution of Myrtaceae using a dated molecular phylogeny. Mol. Phylogenet Evol..

[54] Xu J, Wang XY, Guo WZ (2015). The cytochrome P450 superfamily: Key players in plant development and defense. JIA..

[55] Rai A, Suprasanna P, D’Souza SF, Kumar V (2012). Membrane Topology and Predicted RNA-Binding Function of the ‘Early Responsive to Dehydration (ERD4)’ Plant Protein. PLoS ONE..

[56] Liu Y (2009). A maize early responsive to dehydration gene, ZmERD4, provides enhanced drought and salt tolerance in *Arabidopsis*. Plant Mol .Biol. Rep..

[57] Qi Z, Xiong L (2013). Characterization of a purine permease family gene OsPUP7 involved in growth and development control in rice. J. Integr. Plant Biol..

