# Supplementary material for: Identification of a molecular marker associated with lignotuber in Eucalyptus ssp
Source: Sci Rep. 2020 Feb 27;10:3608. doi: 10.1038/s41598-020-60308-8 (PMC7046637; doi:10.1038/s41598-020-60308-8)
Supplement: Supplementary file 1 — Supplementary material. [file 41598_2020_60308_MOESM1_ESM.pdf]

Identification of a molecular marker associated with lignotuber in *Eucalyptus ssp.*

Tânia M. Bortoloto[1], Maria C. P. Fuchs-Ferraz[1], Karine Kettener[1], Lígia Martins Rubio[1], Esteban R. González[2], Izabel C. G. de Souza[2], Shinitiro Oda[2], Bruno C. Rossini[1,3*], Celso L. Marino[1,3]

[1]Departamento de Genética, Instituto de Biociências, UNESP - Univ Estadual Paulista, R. Prof. Dr. Antônio Celso Wagner Zanin s/n, Botucatu, SP CEP 18618-689, Brazil.

[2]Suzano Papel e Celulose SA, Av. Dr. José Lembo 1010, Itapetininga, SP CEP 18207-780, Brazil.

[3]Instituto de Biotecnologia (IBTEC), UNESP - Univ Estadual Paulista, Alameda das Tecomarias s/n, Botucatu, SP CEP 18607-440, Brazil.

Supplementary Figure 1. F2 cross individuals (hybrids of *Eucalyptus grandi*s x *E. urophylla*) submitted to hydric and nutrient stress in greenhouse. The evaluation after 12 months showed a 3:1 ratio (with lignotuber ; without lignotuber) as showed by qui-square test.

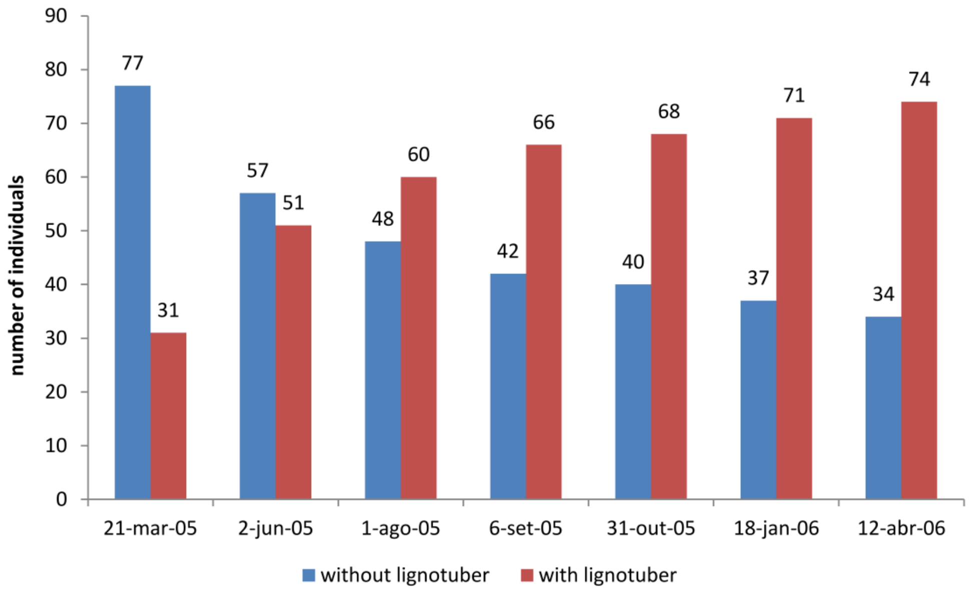

Supplementary Figure 2. *ELig* marker profiles in thirteen individuals of *E. grandis* and *E. urophylla*, showing bands in species without the lignotuber phenotype. Molecular size marker ladder (M), C-: Negative control.

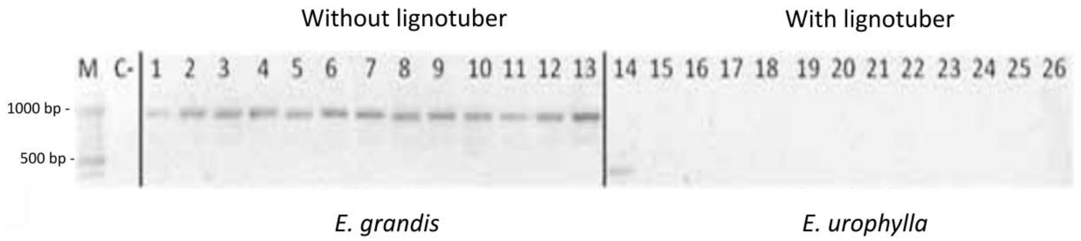

Supplementary Figure 3. Southern blot technique with DNA of *E. grandis* (G) and *E. urophylla* (U) digested with restriction enzymes *EcoR*I, *Nde*I, and *Xba*I, showing a single copy of the *ELig* marker in the species.

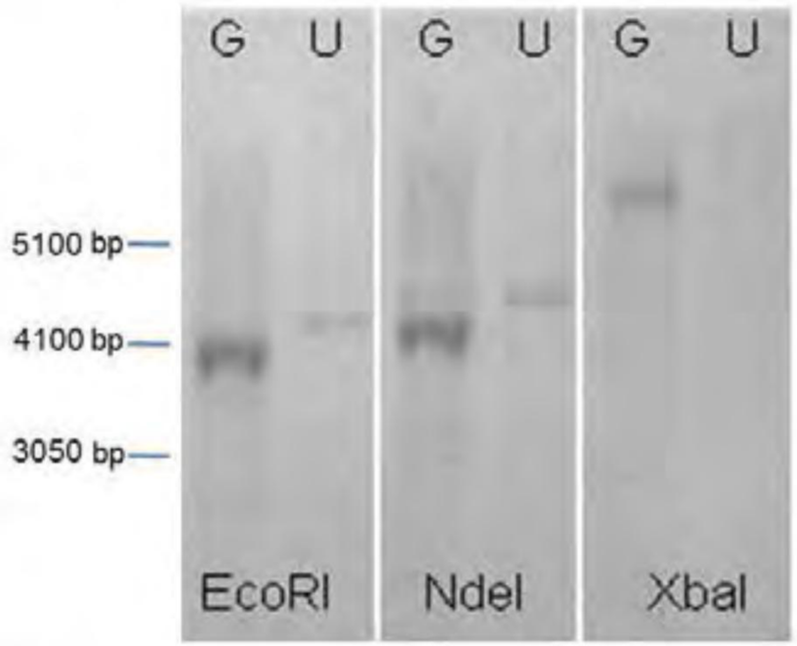

Supplementary Table 1. GO analysis in genes surrounding *Elig* sequence. It was found 125 genes in an interval of 2.6Mb.

SeqName	Phytozome description	GO IDs	GO Names	Enzyme Codes	Enzyme Names	InterPro IDs	InterPro GO IDs	InterPro GO Names
Eucgr.D00196	K14427 - solute carrier family 12 (potassium/chloride transporter), member 4/5/6 (SLC12A4_5_6, KCC) (1 of 1) OS=Egrandis	F:GO:0015377; C:GO:0016021; P:GO:1902476	F:cation:chloride symporter activity; C:integral component of membrane; P:chloride transmembrane transport			IPR018491 (PFAM); PTHR11827 (PANTHER); PTHR11827:SF68 (PANTHER)	F:GO:0005215; P:GO:0006811; C:GO:0016020	F:transporter activity; P:ion transport; C:membrane
Eucgr.D00197	PF00651//PF03000 - BTB/POZ domain (BTB) // NPH3 family (NPH3) (1 of 16) OS=Egrandis	P:GO:0016567	P:protein ubiquitination			IPR027356 (PFAM); PTHR32370:SF12 (PANTHER); PTHR32370 (PANTHER); IPR027356 (PROSITE_PROFILES); IPR011333 (SUPERFAMILY)	no GO terms	no GO terms
Eucgr.D00198	uncharacterized protein OS=Egrandis					no IPS match	no IPS match	no IPS match
Eucgr.D00200	KOG0156 - Cytochrome P450 CYP2 subfamily (1 of 230) OS=Egrandis	F:GO:0004497; F:GO:0005506; C:GO:0016021; F:GO:0016705; F:GO:0020037; P:GO:0055114	F:monooxygenase activity; F:iron ion binding; C:integral component of membrane; F:oxidoreductase activity, acting on paired donors, with incorporation or reduction of molecular oxygen; F:heme binding; P:oxidation-reduction process			IPR002401 (PRINTS); IPR001128 (PFAM); IPR036396 (G3DSA:1.10.630.GENE3D); PTHR24298:SF312 (PANTHER); PTHR24298 (PANTHER); IPR036396 (SUPERFAMILY)	F:GO:0005506; F:GO:0016705; F:GO:0020037; P:GO:0055114	F:iron ion binding; F:oxidoreductase activity, acting on paired donors, with incorporation or reduction of molecular oxygen; F:heme binding; P:oxidation-reduction process
Eucgr.D00201	KOG0156 - Cytochrome P450 CYP2 subfamily (1 of 230) OS=Egrandis	F:GO:0004497; F:GO:0005506; C:GO:0016021; F:GO:0016705; F:GO:0020037; P:GO:0055114	F:monooxygenase activity; F:iron ion binding; C:integral component of membrane; F:oxidoreductase activity, acting on paired donors, with incorporation or reduction of molecular oxygen; F:heme binding; P:oxidation-reduction process			IPR002401 (PRINTS); IPR001128 (PFAM); IPR036396 (G3DSA:1.10.630.GENE3D); PTHR24298 (PANTHER); PTHR24298:SF312 (PANTHER); cd12087 (CDD); IPR036396 (SUPERFAMILY)	F:GO:0005506; F:GO:0016705; F:GO:0020037; P:GO:0055114	F:iron ion binding; F:oxidoreductase activity, acting on paired donors, with incorporation or reduction of molecular oxygen; F:heme binding; P:oxidation-reduction process
Eucgr.D00203	KOG0156 - Cytochrome P450 CYP2 subfamily (1 of 230) OS=Egrandis	F:GO:0004497; F:GO:0005506; C:GO:0016021; F:GO:0016705; F:GO:0020037; P:GO:0055114	F:monooxygenase activity; F:iron ion binding; C:integral component of membrane; F:oxidoreductase activity, acting on paired donors, with incorporation or reduction of molecular oxygen; F:heme binding; P:oxidation-reduction process			IPR002401 (PRINTS); IPR001128 (PFAM); IPR036396 (G3DSA:1.10.630.GENE3D); PTHR24298 (PANTHER); PTHR24298:SF312 (PANTHER); IPR036396 (SUPERFAMILY)	F:GO:0005506; F:GO:0016705; F:GO:0020037; P:GO:0055114	F:iron ion binding; F:oxidoreductase activity, acting on paired donors, with incorporation or reduction of molecular oxygen; F:heme binding; P:oxidation-reduction process
Eucgr.D00205	KOG0156 - Cytochrome P450 CYP2 subfamily (1 of 230) OS=Egrandis	F:GO:0004497; F:GO:0005506; C:GO:0016021; F:GO:0016705; F:GO:0020037; P:GO:0055114	F:monooxygenase activity; F:iron ion binding; C:integral component of membrane; F:oxidoreductase activity, acting on paired donors, with incorporation or reduction of molecular oxygen; F:heme binding; P:oxidation-reduction process			IPR002401 (PRINTS); IPR001128 (PFAM); IPR036396 (G3DSA:1.10.630.GENE3D); PTHR24298 (PANTHER); PTHR24298:SF312 (PANTHER); IPR036396 (SUPERFAMILY)	F:GO:0005506; F:GO:0016705; F:GO:0020037; P:GO:0055114	F:iron ion binding; F:oxidoreductase activity, acting on paired donors, with incorporation or reduction of molecular oxygen; F:heme binding; P:oxidation-reduction process
Eucgr.D00206	KOG0156 - Cytochrome P450 CYP2 subfamily (1 of 230) OS=Egrandis	F:GO:0004497; F:GO:0005506; C:GO:0016021; F:GO:0016705; F:GO:0020037; P:GO:0055114	F:monooxygenase activity; F:iron ion binding; C:integral component of membrane; F:oxidoreductase activity, acting on paired donors, with incorporation or reduction of molecular oxygen; F:heme binding; P:oxidation-reduction process			IPR002401 (PRINTS); IPR036396 (G3DSA:1.10.630.GENE3D); IPR001128 (PFAM); PTHR24298 (PANTHER); PTHR24298:SF312 (PANTHER); IPR036396 (SUPERFAMILY)	F:GO:0005506; F:GO:0016705; F:GO:0020037; P:GO:0055114	F:iron ion binding; F:oxidoreductase activity, acting on paired donors, with incorporation or reduction of molecular oxygen; F:heme binding; P:oxidation-reduction process
Eucgr.D00207	PF13961 - Domain of unknown function (DUF4219) (DUF4219) (1 of 14) OS=Egrandis					PF14223 (PFAM); IPR025314 (PFAM)	no GO terms	no GO terms
Eucgr.D00208	PF00067 - Cytochrome P450 (p450) (1 of 572) OS=Egrandis	F:GO:0004497; F:GO:0005506; C:GO:0016021; F:GO:0016705; F:GO:0020037; P:GO:0055114	F:monooxygenase activity; F:iron ion binding; C:integral component of membrane; F:oxidoreductase activity, acting on paired donors, with incorporation or reduction of molecular oxygen; F:heme binding; P:oxidation-reduction process			PTHR24298 (PANTHER); PTHR24298:SF136 (PANTHER); IPR036396 (SUPERFAMILY)	F:GO:0005506; F:GO:0016705; F:GO:0020037; P:GO:0055114	F:iron ion binding; F:oxidoreductase activity, acting on paired donors, with incorporation or reduction of molecular oxygen; F:heme binding; P:oxidation-reduction process
Eucgr.D00209	KOG0156 - Cytochrome P450 CYP2 subfamily (1 of 230) OS=Egrandis	F:GO:0004497; F:GO:0005506; C:GO:0016021; F:GO:0016705; F:GO:0020037; P:GO:0055114	F:monooxygenase activity; F:iron ion binding; C:integral component of membrane; F:oxidoreductase activity, acting on paired donors, with incorporation or reduction of molecular oxygen; F:heme binding; P:oxidation-reduction process			IPR001128 (PFAM); IPR036396 (G3DSA:1.10.630.GENE3D); PTHR24298:SF312 (PANTHER); PTHR24298 (PANTHER); IPR036396 (SUPERFAMILY)	F:GO:0005506; F:GO:0016705; F:GO:0020037; P:GO:0055114	F:iron ion binding; F:oxidoreductase activity, acting on paired donors, with incorporation or reduction of molecular oxygen; F:heme binding; P:oxidation-reduction process
Eucgr.D00210	PF00067 - Cytochrome P450 (p450) (1 of 572) OS=Egrandis	F:GO:0004497; F:GO:0005506; C:GO:0016021; F:GO:0016705; F:GO:0020037; P:GO:0055114	F:monooxygenase activity; F:iron ion binding; C:integral component of membrane; F:oxidoreductase activity, acting on paired donors, with incorporation or reduction of molecular oxygen; F:heme binding; P:oxidation-reduction process			IPR002401 (PRINTS); IPR001128 (PRINTS); IPR036396 (G3DSA:1.10.630.GENE3D); IPR001128 (PFAM); PTHR24298 (PANTHER); PTHR24298:SF136 (PANTHER); IPR036396 (SUPERFAMILY)	F:GO:0005506; F:GO:0016705; F:GO:0020037; P:GO:0055114	F:iron ion binding; F:oxidoreductase activity, acting on paired donors, with incorporation or reduction of molecular oxygen; F:heme binding; P:oxidation-reduction process
Eucgr.D00211	PF00067 - Cytochrome P450 (p450) (1 of 572) OS=Egrandis	F:GO:0004497; F:GO:0005506; C:GO:0016021; F:GO:0016705; F:GO:0020037; P:GO:0055114	F:monooxygenase activity; F:iron ion binding; C:integral component of membrane; F:oxidoreductase activity, acting on paired donors, with incorporation or reduction of molecular oxygen; F:heme binding; P:oxidation-reduction process			IPR036396 (G3DSA:1.10.630.GENE3D); IPR001128 (PFAM); PTHR24298 (PANTHER); PTHR24298:SF312 (PANTHER); IPR036396 (SUPERFAMILY)	F:GO:0005506; F:GO:0016705; F:GO:0020037; P:GO:0055114	F:iron ion binding; F:oxidoreductase activity, acting on paired donors, with incorporation or reduction of molecular oxygen; F:heme binding; P:oxidation-reduction process
Eucgr.D00212	uncharacterized protein OS=Egrandis					mobidb-lite (MOBIDB_LITE); mobidb-lite (MOBIDB_LITE)	no GO terms	no GO terms
Eucgr.D00213	KOG0156 - Cytochrome P450 CYP2 subfamily (1 of 230) OS=Egrandis	F:GO:0004497; F:GO:0005506; C:GO:0016021; F:GO:0016705; F:GO:0020037; P:GO:0055114	F:monooxygenase activity; F:iron ion binding; C:integral component of membrane; F:oxidoreductase activity, acting on paired donors, with incorporation or reduction of molecular oxygen; F:heme binding; P:oxidation-reduction process			IPR002401 (PRINTS); IPR001128 (PFAM); IPR036396 (G3DSA:1.10.630.GENE3D); PTHR24298:SF312 (PANTHER); PTHR24298 (PANTHER); IPR036396 (SUPERFAMILY)	F:GO:0005506; F:GO:0016705; F:GO:0020037; P:GO:0055114	F:iron ion binding; F:oxidoreductase activity, acting on paired donors, with incorporation or reduction of molecular oxygen; F:heme binding; P:oxidation-reduction process
Eucgr.D00214	KOG0156 - Cytochrome P450 CYP2 subfamily (1 of 230) OS=Egrandis	F:GO:0004497; F:GO:0005506; C:GO:0016021; F:GO:0016705; F:GO:0020037; P:GO:0055114	F:monooxygenase activity; F:iron ion binding; C:integral component of membrane; F:oxidoreductase activity, acting on paired donors, with incorporation or reduction of molecular oxygen; F:heme binding; P:oxidation-reduction process			IPR002401 (PRINTS); IPR036396 (G3DSA:1.10.630.GENE3D); IPR001128 (PFAM); PTHR24298:SF312 (PANTHER); PTHR24298 (PANTHER); IPR036396 (SUPERFAMILY)	F:GO:0005506; F:GO:0016705; F:GO:0020037; P:GO:0055114	F:iron ion binding; F:oxidoreductase activity, acting on paired donors, with incorporation or reduction of molecular oxygen; F:heme binding; P:oxidation-reduction process
Eucgr.D00215	PTHR24223:SF251 - ABC TRANSPORTER C FAMILY MEMBER 11-RELATED (1 of 15) OS=Egrandis	F:GO:0005524; C:GO:0016021; F:GO:0042626; P:GO:0055085	F:ATP binding; C:integral component of membrane; F:ATPase activity, coupled to transmembrane movement of substances; P:transmembrane transport	EC:3.6.1.3; EC:3.6.1.15	Adenosinetriphosphatase; Nucleoside-triphosphate phosphatase	IPR036640 (G3DSA:1.20.1560.GENE3D); IPR003439 (PFAM); G3DSA:3.40.50.300 (GENE3D); PTHR24223:SF251 (PANTHER); PTHR24223 (PANTHER); IPR011527 (PROSITE_PROFILES); IPR036640 (SUPERFAMILY); IPR027417 (SUPERFAMILY)	F:GO:0005524; C:GO:0016021; F:GO:0016887; F:GO:0042626; P:GO:0055085	F:ATP binding; C:integral component of membrane; F:ATPase activity; F:ATPase activity, coupled to transmembrane movement of substances; P:transmembrane transport
Eucgr.D00216	5.99.1.2 - DNA topoisomerase / Untwisting enzyme (1 of 35) OS=Egrandis	F:GO:0008270; C:GO:0016021	F:zinc ion binding; C:integral component of membrane			IPR010666 (PFAM); PTHR33248 (PANTHER)	F:GO:0008270	F:zinc ion binding
Eucgr.D00217	KOG0058 - Peptide exporter, ABC superfamily (1 of 9) OS=Egrandis	F:GO:0005524; C:GO:0016021; F:GO:0042626; P:GO:0055085	F:ATP binding; C:integral component of membrane; F:ATPase activity, coupled to transmembrane movement of substances; P:transmembrane transport	EC:3.6.1.3; EC:3.6.1.15	Adenosinetriphosphatase; Nucleoside-triphosphate phosphatase	IPR036640 (G3DSA:1.20.1560.GENE3D); IPR003439 (PFAM); G3DSA:3.40.50.300 (GENE3D); PTHR24223:SF251 (PANTHER); PTHR24223 (PANTHER); IPR011527 (PROSITE_PROFILES); IPR027417 (SUPERFAMILY); IPR036640 (SUPERFAMILY)	F:GO:0005524; C:GO:0016021; F:GO:0016887; F:GO:0042626; P:GO:0055085	F:ATP binding; C:integral component of membrane; F:ATPase activity; F:ATPase activity, coupled to transmembrane movement of substances; P:transmembrane transport
Eucgr.D00218	PTHR24223:SF251 - ABC TRANSPORTER C FAMILY MEMBER 11-RELATED (1 of 15) OS=Egrandis	F:GO:0005524; C:GO:0016021; F:GO:0042626; P:GO:0055085	F:ATP binding; C:integral component of membrane; F:ATPase activity, coupled to transmembrane movement of substances; P:transmembrane transport	EC:3.6.1.3; EC:3.6.1.15	Adenosinetriphosphatase; Nucleoside-triphosphate phosphatase	PTHR24223 (PANTHER); PTHR24223:SF251 (PANTHER)	no GO terms	no GO terms
Eucgr.D00219	KOG0058 - Peptide exporter, ABC superfamily (1 of 9) OS=Egrandis	F:GO:0005524; C:GO:0016021; F:GO:0042626; P:GO:0055085	F:ATP binding; C:integral component of membrane; F:ATPase activity, coupled to transmembrane movement of substances; P:transmembrane transport	EC:3.6.1.3; EC:3.6.1.15	Adenosinetriphosphatase; Nucleoside-triphosphate phosphatase	G3DSA:3.40.50.300 (GENE3D); IPR003439 (PFAM); IPR036640 (G3DSA:1.20.1560.GENE3D); PTHR24223:SF251 (PANTHER); PTHR24223 (PANTHER); IPR011527 (PROSITE_PROFILES); IPR036640 (SUPERFAMILY); IPR027417 (SUPERFAMILY)	F:GO:0005524; C:GO:0016021; F:GO:0016887; F:GO:0042626; P:GO:0055085	F:ATP binding; C:integral component of membrane; F:ATPase activity; F:ATPase activity, coupled to transmembrane movement of substances; P:transmembrane transport
Eucgr.D00220	PTHR24223:SF251 - ABC TRANSPORTER C FAMILY MEMBER 11-RELATED (1 of 15) OS=Egrandis	F:GO:0005524; C:GO:0016021; F:GO:0042626; P:GO:0055085	F:ATP binding; C:integral component of membrane; F:ATPase activity, coupled to transmembrane movement of substances; P:transmembrane transport	EC:3.6.1.3; EC:3.6.1.15	Adenosinetriphosphatase; Nucleoside-triphosphate phosphatase	PTHR24223 (PANTHER); PTHR24223:SF251 (PANTHER)	no GO terms	no GO terms
Eucgr.D00222	PF01535//PF12854//PF13041 - PPR repeat (PPR) // PPR repeat (PPR_1) // PPR repeat family (PPR_2) (1 of 33) OS=Egrandis	F:GO:0005515	F:protein binding			(G3DSA:1.25.40.GENE3D); IPR011990 (G3DSA:1.25.40.GENE3D); IPR002885 (PFAM); IPR011990 (G3DSA:1.25.40.GENE3D); IPR002885 (PFAM); IPR002885 (TIGRFAM); PTHR24015 (PANTHER); PTHR24015:SF656 (PANTHER); IPR002885 (PROSITE_PROFILES); IPR002885 (PROSITE_PROFILES); IPR002885 (PROSITE_PROFILES); IPR002885 (PROSITE_PROFILES); IPR002885 (PROSITE_PROFILES); IPR002885 (PROSITE_PROFILES); IPR002885 (PROSITE_PROFILES); IPR002885 (PROSITE_PROFILES); IPR002885 (PROSITE_PROFILES); IPR002885 (PROSITE_PROFILES); IPR002885 (PROSITE_PROFILES); IPR002885 (PROSITE_PROFILES); IPR002885 (PROSITE_PROFILES); IPR002885 (PROSITE_PROFILES); IPR002885 (PROSITE_PROFILES); IPR002885 (PROSITE_PROFILES); IPR002885 (PROSITE_PROFILES); IPR002885 (PROSITE_PROFILES); IPR002885 (PROSITE_PROFILES);	F:GO:0005515	F:protein binding

Eucgr.D00223	PTHR24223:SF251 - ABC TRANSPORTER C FAMILY MEMBER 11-RELATED (1 of 15) OS=Egrandis	F:GO:0003676; F:GO:0005524; F:GO:0008270; C:GO:0016021; F:GO:0042626; P:GO:0055085	F:nucleic acid binding; F:ATP binding; F:zinc ion binding; C:integral component of membrane; F:ATPase activity, coupled to transmembrane movement of substances; P:transmembrane transport	EC:3.6.1.3; EC:3.6.1.15	Adenosinetriphosphatase; Nucleoside-triphosphate phosphatase	IPR003439 (PFAM); G3DSA:3.40.50.300 (GENE3D); IPR036640 (G3DSA:1.20.1560.GENE3D); PTHR24223:SF251 (PANTHER); PTHR24223 (PANTHER); IPR011527 (PROSITE_PROFILES); IPR027417 (SUPERFAMILY); IPR036640 (SUPERFAMILY)	F:GO:0005524; C:GO:0016021; F:GO:0016887; F:GO:0042626; P:GO:0055085	F:ATP binding; C:integral component of membrane; F:ATPase activity; F:ATPase activity, coupled to transmembrane movement of substances; P:transmembrane transport
Eucgr.D00224	PTHR24223:SF251 - ABC TRANSPORTER C FAMILY MEMBER 11-RELATED (1 of 15) OS=Egrandis	F:GO:0005524; C:GO:0016021; F:GO:0042626; P:GO:0055085	F:ATP binding; C:integral component of membrane; F:ATPase activity, coupled to transmembrane movement of substances; P:transmembrane transport	EC:3.6.1.3; EC:3.6.1.15	Adenosinetriphosphatase; Nucleoside-triphosphate phosphatase	IPR036640 (G3DSA:1.20.1560.GENE3D); G3DSA:3.40.50.300 (GENE3D); IPR003439 (PFAM); PTHR24223 (PANTHER); PTHR24223:SF251 (PANTHER); IPR011527 (PROSITE_PROFILES); IPR027417 (SUPERFAMILY); IPR036640 (SUPERFAMILY)	F:GO:0005524; C:GO:0016021; F:GO:0016887; F:GO:0042626; P:GO:0055085	F:ATP binding; C:integral component of membrane; F:ATPase activity; F:ATPase activity, coupled to transmembrane movement of substances; P:transmembrane transport
Eucgr.D00225	PTHR24223:SF251 - ABC TRANSPORTER C FAMILY MEMBER 11-RELATED (1 of 15) OS=Egrandis	F:GO:0005524; C:GO:0016021; F:GO:0042626; P:GO:0055085	F:ATP binding; C:integral component of membrane; F:ATPase activity, coupled to transmembrane movement of substances; P:transmembrane transport	EC:3.6.1.3; EC:3.6.1.15	Adenosinetriphosphatase; Nucleoside-triphosphate phosphatase	PTHR24223 (PANTHER); PTHR24223:SF251 (PANTHER)	no GO terms	no GO terms
Eucgr.D00226	PTHR24223:SF251 - ABC TRANSPORTER C FAMILY MEMBER 11-RELATED (1 of 15) OS=Egrandis	F:GO:0005524; C:GO:0016021; F:GO:0042626; P:GO:0055085	F:ATP binding; C:integral component of membrane; F:ATPase activity, coupled to transmembrane movement of substances; P:transmembrane transport	EC:3.6.1.3; EC:3.6.1.15	Adenosinetriphosphatase; Nucleoside-triphosphate phosphatase	IPR003439 (PFAM); G3DSA:3.40.50.300 (GENE3D); PTHR24223:SF251 (PANTHER); PTHR24223 (PANTHER); IPR027417 (SUPERFAMILY)	F:GO:0005524; F:GO:0016887	F:ATP binding; F:ATPase activity
Eucgr.D00228	PTHR24223:SF251 - ABC TRANSPORTER C FAMILY MEMBER 11-RELATED (1 of 15) OS=Egrandis	F:GO:0005524; C:GO:0016021; F:GO:0042626; P:GO:0055085	F:ATP binding; C:integral component of membrane; F:ATPase activity, coupled to transmembrane movement of substances; P:transmembrane transport	EC:3.6.1.3; EC:3.6.1.15	Adenosinetriphosphatase; Nucleoside-triphosphate phosphatase	IPR036640 (G3DSA:1.20.1560.GENE3D); IPR003439 (PFAM); G3DSA:3.40.50.300 (GENE3D); PTHR24223:SF251 (PANTHER); PTHR24223 (PANTHER); IPR011527 (PROSITE_PROFILES); IPR027417 (SUPERFAMILY); IPR036640 (SUPERFAMILY)	F:GO:0005524; C:GO:0016021; F:GO:0016887; F:GO:0042626; P:GO:0055085	F:ATP binding; C:integral component of membrane; F:ATPase activity; F:ATPase activity, coupled to transmembrane movement of substances; P:transmembrane transport
Eucgr.D00230	PTHR24223:SF251 - ABC TRANSPORTER C FAMILY MEMBER 11-RELATED (1 of 15) OS=Egrandis	F:GO:0005524; C:GO:0016021; F:GO:0042626; P:GO:0055085	F:ATP binding; C:integral component of membrane; F:ATPase activity, coupled to transmembrane movement of substances; P:transmembrane transport	EC:3.6.1.3; EC:3.6.1.15	Adenosinetriphosphatase; Nucleoside-triphosphate phosphatase	IPR036640 (G3DSA:1.20.1560.GENE3D); IPR003439 (PFAM); G3DSA:3.40.50.300 (GENE3D); PTHR24223:SF251 (PANTHER); PTHR24223 (PANTHER); IPR011527 (PROSITE_PROFILES); IPR027417 (SUPERFAMILY); IPR036640 (SUPERFAMILY)	F:GO:0005524; C:GO:0016021; F:GO:0016887; F:GO:0042626; P:GO:0055085	F:ATP binding; C:integral component of membrane; F:ATPase activity; F:ATPase activity, coupled to transmembrane movement of substances; P:transmembrane transport
Eucgr.D00231	PTHR24223:SF251 - ABC TRANSPORTER C FAMILY MEMBER 11-RELATED (1 of 15) OS=Egrandis	F:GO:0005524; C:GO:0016021; F:GO:0042626; P:GO:0055085	F:ATP binding; C:integral component of membrane; F:ATPase activity, coupled to transmembrane movement of substances; P:transmembrane transport	EC:3.6.1.3; EC:3.6.1.15	Adenosinetriphosphatase; Nucleoside-triphosphate phosphatase	PTHR24223:SF251 (PANTHER); PTHR24223 (PANTHER)	no GO terms	no GO terms
Eucgr.D00232	uncharacterized protein OS=Egrandis	C:GO:0016021	C:integral component of membrane			no IPS match	no IPS match	no IPS match
Eucgr.D00233	PTHR24223:SF251 - ABC TRANSPORTER C FAMILY MEMBER 11-RELATED (1 of 15) OS=Egrandis	F:GO:0005524; C:GO:0016021; F:GO:0042626; P:GO:0055085	F:ATP binding; C:integral component of membrane; F:ATPase activity, coupled to transmembrane movement of substances; P:transmembrane transport	EC:3.6.1.3; EC:3.6.1.15	Adenosinetriphosphatase; Nucleoside-triphosphate phosphatase	G3DSA:3.40.50.300 (GENE3D); IPR003439 (PFAM); IPR036640 (G3DSA:1.20.1560.GENE3D); PTHR24223:SF251 (PANTHER); PTHR24223 (PANTHER); IPR011527 (PROSITE_PROFILES); IPR036640 (SUPERFAMILY); IPR027417 (SUPERFAMILY)	F:GO:0005524; C:GO:0016021; F:GO:0016887; F:GO:0042626; P:GO:0055085	F:ATP binding; C:integral component of membrane; F:ATPase activity; F:ATPase activity, coupled to transmembrane movement of substances; P:transmembrane transport
Eucgr.D00234	K02698 - photosystem I subunit X (psaK) (1 of 1) OS=Egrandis	C:GO:0009522; P:GO:0015979; C:GO:0016021	C:photosystem I; P:photosynthesis; C:integral component of membrane			IPR017493 (TIGRFAM); IPR000549 (PFAM); IPR023618 (G3DSA:1.10.286.GENE3D); PTHR34195 (PANTHER); PTHR34195:SF2 (PANTHER); IPR035982 (SUPERFAMILY)	C:GO:0009522; P:GO:0015979; C:GO:0016020	C:photosystem I; P:photosynthesis; C:membrane
Eucgr.D00235	2.7.11.1 - Non-specific serine/threonine protein kinase / Threonine-specific protein kinase (1 of 2412) OS=Egrandis	F:GO:0016301	F:kinase activity			no IPS match	no IPS match	no IPS match
Eucgr.D00236	PF01535//PF13041 - PPR repeat (PPR) // PPR repeat family (PPR_2) (1 of 235) OS=Egrandis	F:GO:0005515	F:protein binding			IPR002885 (TIGRFAM); IPR011990 (G3DSA:1.25.40.GENE3D); IPR002885 (PFAM); IPR002885 (PFAM); IPR011990 (G3DSA:1.25.40.GENE3D); IPR011990 (G3DSA:1.25.40.GENE3D); PTHR24015 (PANTHER); PTHR24015:SF535 (PANTHER); IPR002885 (PROSITE_PROFILES); IPR002885 (PROSITE_PROFILES); IPR002885 (PROSITE_PROFILES); IPR002885 (PROSITE_PROFILES); IPR002885 (PROSITE_PROFILES); IPR002885 (PROSITE_PROFILES); IPR002885 (PROSITE_PROFILES); IPR002885 (PROSITE_PROFILES); IPR002885 (PROSITE_PROFILES); IPR002885 (PROSITE_PROFILES)	F:GO:0005515	F:protein binding
Eucgr.D00237	PF00069//PF08263//PF13855 - Protein kinase domain (Pkinase) // Leucine rich repeat N-terminal domain (LRRNT_2) // Leucine rich repeat (LRR_8) (1 of 79) OS=Egrandis	F:GO:0004674; F:GO:0005515; F:GO:0005524; P:GO:0006468; C:GO:0016021	F:protein serine/threonine kinase activity; F:protein binding; F:ATP binding; P:protein phosphorylation; C:integral component of membrane	EC:2.7.11	Transferring phosphorus-containing groups	IPR003591 (SMART); IPR000719 (SMART); IPR001611 (PFAM); IPR032675 (G3DSA:3.80.10.GENE3D); G3DSA:1.10.510.10 (GENE3D); IPR000719 (PFAM); IPR001611 (PFAM); PTHR43887:SF22 (PANTHER); PTHR43887 (PANTHER); IPR000719 (PROSITE_PROFILES); SSF52058 (SUPERFAMILY); SSF52058 (SUPERFAMILY); IPR011009 (SUPERFAMILY)	F:GO:0004672; F:GO:0005515; F:GO:0005524; P:GO:0006468	F:protein kinase activity; F:protein binding; F:ATP binding; P:protein phosphorylation
Eucgr.D00239	PF00069//PF00560//PF08263//PF13855 - Protein kinase domain (Pkinase) // Leucine Rich Repeat (LRR_1) // Leucine rich repeat N-terminal domain (LRRNT_2) // Leucine rich repeat (LRR_8) (1 of 110) OS=Egrandis	F:GO:0004674; F:GO:0005515; F:GO:0005524; P:GO:0006468; C:GO:0016021	F:protein serine/threonine kinase activity; F:protein binding; F:ATP binding; P:protein phosphorylation; C:integral component of membrane	EC:2.7.11	Transferring phosphorus-containing groups	IPR000719 (SMART); IPR003591 (SMART); G3DSA:1.10.510.10 (GENE3D); IPR032675 (G3DSA:3.80.10.GENE3D); IPR032675 (G3DSA:3.80.10.GENE3D); IPR001611 (PFAM); IPR001611 (PFAM); IPR000719 (PFAM); IPR032675 (G3DSA:3.80.10.GENE3D); PTHR43887 (PANTHER); PTHR43887:SF22 (PANTHER); IPR000719 (PROSITE_PROFILES); SSF52058 (SUPERFAMILY); IPR011009 (SUPERFAMILY); SSF52058 (SUPERFAMILY)	F:GO:0004672; F:GO:0005515; F:GO:0005524; P:GO:0006468	F:protein kinase activity; F:protein binding; F:ATP binding; P:protein phosphorylation
Eucgr.D00241	PF07714//PF08263//PF13855 - Protein tyrosine kinase (Pkinase_Tyr) // Leucine rich repeat N-terminal domain (LRRNT_2) // Leucine rich repeat (LRR_8) (1 of 29) OS=Egrandis	F:GO:0004674; F:GO:0005515; F:GO:0005524; P:GO:0006468; C:GO:0016021	F:protein serine/threonine kinase activity; F:protein binding; F:ATP binding; P:protein phosphorylation; C:integral component of membrane	EC:2.7.11	Transferring phosphorus-containing groups	IPR003591 (SMART); IPR000719 (SMART); G3DSA:1.10.510.10 (GENE3D); IPR032675 (G3DSA:3.80.10.GENE3D); IPR000719 (PFAM); IPR032675 (G3DSA:3.80.10.GENE3D); IPR001611 (PFAM); PTHR43887:SF22 (PANTHER); PTHR43887 (PANTHER); IPR000719 (PROSITE_PROFILES); SSF52058 (SUPERFAMILY); IPR011009 (SUPERFAMILY); SSF52058 (SUPERFAMILY)	F:GO:0004672; F:GO:0005515; F:GO:0005524; P:GO:0006468	F:protein kinase activity; F:protein binding; F:ATP binding; P:protein phosphorylation
Eucgr.D00242	K10683 - BRCA1-associated RING domain protein 1 [EC:6.3.2.19] (BARD1) (1 of 1) OS=Egrandis	P:GO:0006281; F:GO:0046872	P:DNA repair; F:metal ion binding			mobidb-lite (MOBIDB_LITE); mobidb-lite (MOBIDB_LITE); mobidb-lite (MOBIDB_LITE); mobidb-lite (MOBIDB_LITE)	no GO terms	no GO terms
Eucgr.D00243	K19371 - DnaJ homolog subfamily C member 25 (DNAJC25) (1 of 1) OS=Egrandis					no IPS match	no IPS match	no IPS match
Eucgr.D00244	uncharacterized protein OS=Egrandis					no IPS match	no IPS match	no IPS match
Eucgr.D00245	PTHR11538:SF40 - PHENYLALANINE--TRNA LIGASE ALPHA SUBUNIT (1 of 1) OS=Egrandis	F:GO:0000049; F:GO:0000166; F:GO:0004812; F:GO:0004826; F:GO:0005524; C:GO:0005737; P:GO:0006432; P:GO:0043039	F:tRNA binding; F:nucleotide binding; F:aminoacyl-tRNA ligase activity; F:phenylalanine-tRNA ligase activity; F:ATP binding; C:cytoplasm; P:phenylalanyl-tRNA aminoacylation; P:tRNA aminoacylation			mobidb-lite (MOBIDB_LITE)	no GO terms	no GO terms

Eucgr.D00246	PTHR12329//PTHR12329:SF17 - BCL2-ASSOCIATED ATHANOGENE // SUBFAMILY NOT NAMED (1 of 1) OS=Egrandis	F:GO:0051087	F:chaperone binding			IPR000626 (PFAM); G3DSA:3.10.20.90 (GENE3D); IPR036533 (G3DSA:1.20.58.GENE3D); mobidb-lite (MOBIDB_LITE); mobidb-lite (MOBIDB_LITE); mobidb-lite (MOBIDB_LITE); PTHR12329:SF17 (PANTHER); IPR039773 (PANTHER); PTHR12329:SF17 (PANTHER); IPR039773 (PANTHER); IPR000626 (PROSITE_PROFILES); IPR029071 (SUPERFAMILY)	F:GO:0005515; F:GO:0051087	F:protein binding; F:chaperone binding
Eucgr.D00247	PTHR22536//PTHR22536:SF2 - LUNG CANCER METASTASIS-RELATED LCMR1 PROTEIN // SUBFAMILY NOT NAMED (1 of 1) OS=Egrandis	C:GO:0016020; C:GO:0016021	C:membrane; C:integral component of membrane			no IPS match	no IPS match	no IPS match
Eucgr.D00248	KOG4615 - Uncharacterized conserved protein (1 of 1) OS=Egrandis	C:GO:0016021	C:integral component of membrane			IPR018614 (PFAM); IPR018614 (PANTHER)	no GO terms	no GO terms
Eucgr.D00249	PTHR11945:SF97 - FLORAL HOMEOTIC PROTEIN PISTILLATA (1 of 3) OS=Egrandis	F:GO:0000977; F:GO:0003700; C:GO:0005634; P:GO:0045944; F:GO:0046983	F:RNA polymerase II regulatory region sequence-specific DNA binding; F:DNA-binding transcription factor activity; C:nucleus; P:positive regulation of transcription by RNA polymerase II; F:protein dimerization activity			no IPS match	no IPS match	no IPS match
Eucgr.D00250	PTHR15922:SF2 - NEUROBLASTOMA-AMPLIFIED SEQUENCE (1 of 2) OS=Egrandis	P:GO:0006890	P:retrograde vesicle-mediated transport, Golgi to endoplasmic reticulum			IPR013244 (PFAM); PTHR15922 (PANTHER); PTHR15922:SF4 (PANTHER)	P:GO:0006890	P:retrograde vesicle-mediated transport, Golgi to endoplasmic reticulum
Eucgr.D00251	PTHR15922:SF2 - NEUROBLASTOMA-AMPLIFIED SEQUENCE (1 of 2) OS=Egrandis					PTHR15922 (PANTHER); PTHR15922:SF4 (PANTHER)	no GO terms	no GO terms
Eucgr.D00253	3.1.1.32 - Phospholipase A(1) / Phospholipase A1 (1 of 24) OS=Egrandis	P:GO:0006629; F:GO:0016787	P:lipid metabolic process; F:hydrolase activity			G3DSA:3.40.50.12520 (GENE3D); IPR002921 (PFAM); PTHR31403 (PANTHER); PTHR31403:SF11 (PANTHER); cd00519 (CDD); IPR029058 (SUPERFAMILY)	P:GO:0006629	P:lipid metabolic process
Eucgr.D00254	PF08488//PF13947 - Wall-associated kinase (WAK) // Wall-associated receptor kinase galacturonan-binding (GUB_WAK_bind) (1 of 8) OS=Egrandis	F:GO:0004674; F:GO:0005524; P:GO:0006468; C:GO:0016021; F:GO:0030247	F:protein serine/threonine kinase activity; F:ATP binding; P:protein phosphorylation; C:integral component of membrane; F:polysaccharide binding	EC:2.7.11	Transferring phosphorus-containing groups	IPR001245 (PFAM); G3DSA:3.30.200.20 (GENE3D); G3DSA:1.10.510.10 (GENE3D); PTHR27005:SF75 (PANTHER); PTHR27005 (PANTHER); IPR000719 (PROSITE_PROFILES); IPR011009 (SUPERFAMILY)	F:GO:0004672; F:GO:0005524; P:GO:0006468	F:protein kinase activity; F:ATP binding; P:protein phosphorylation
Eucgr.D00258	1.1.1.31 - 3-hydroxyisobutyrate dehydrogenase (1 of 2) OS=Egrandis	P:GO:0006574; F:GO:0008442; C:GO:0016021; F:GO:0050661; F:GO:0051287; P:GO:0055114	P:valine catabolic process; F:3-hydroxyisobutyrate dehydrogenase activity; C:integral component of membrane; F:NADP binding; F:NAD binding; P:oxidation-reduction process	EC:1.1.1.31	3-hydroxyisobutyrate dehydrogenase	mobidb-lite (MOBIDB_LITE); mobidb-lite (MOBIDB_LITE)	no GO terms	no GO terms
Eucgr.D00260	PTHR13018:SF22 - EARLY-RESPONSIVE TO DEHYDRATION STRESS PROTEIN (ERD4) (1 of 2) OS=Egrandis	C:GO:0016021	C:integral component of membrane			IPR003864 (PFAM); IPR027815 (PFAM); PTHR13018:SF22 (PANTHER); PTHR13018 (PANTHER)	C:GO:0016020	C:membrane
Eucgr.D00261	PTHR13018:SF22 - EARLY-RESPONSIVE TO DEHYDRATION STRESS PROTEIN (ERD4) (1 of 2) OS=Egrandis	C:GO:0016021	C:integral component of membrane			IPR003864 (PFAM); IPR027815 (PFAM); PTHR13018:SF22 (PANTHER); PTHR13018 (PANTHER)	C:GO:0016020	C:membrane
Eucgr.D00262	uncharacterized protein OS=Egrandis	C:GO:0005634; P:GO:0006355; C:GO:0009507; P:GO:0090228	C:nucleus; P:regulation of transcription, DNA-templated; C:chloroplast; P:positive regulation of red or far-red light signaling pathway			mobidb-lite (MOBIDB_LITE); mobidb-lite (MOBIDB_LITE); mobidb-lite (MOBIDB_LITE); IPR034581 (PANTHER)	C:GO:0005634; P:GO:0006355; P:GO:0009416; C:GO:0009507; P:GO:0090228	C:nucleus; P:regulation of transcription, DNA-templated; P:response to light stimulus; C:chloroplast; P:positive regulation of red or far-red light signaling pathway
Eucgr.D00263	K08272 - calcium binding protein 39 (CAB39, MO25) (1 of 2) OS=Egrandis					no IPS match	no IPS match	no IPS match
Eucgr.D00264	PF02362//PF06507 - B3 DNA binding domain (B3) // Auxin response factor (Auxin_resp) (1 of 7) OS=Egrandis	F:GO:0003677; C:GO:0005634; P:GO:0006355; P:GO:0009734	F:DNA binding; C:nucleus; P:regulation of transcription, DNA-templated; P:auxin-activated signaling pathway			mobidb-lite (MOBIDB_LITE); mobidb-lite (MOBIDB_LITE); mobidb-lite (MOBIDB_LITE); PTHR31384 (PANTHER); PTHR31384:SF68 (PANTHER)	no GO terms	no GO terms
Eucgr.D00265	PF03763 - Remorin, C-terminal region (Remorin_C) (1 of 18) OS=Egrandis					PTHR31471 (PANTHER); PTHR31471:SF17 (PANTHER)	no GO terms	no GO terms
Eucgr.D00266	uncharacterized protein OS=Egrandis					PTHR38226 (PANTHER)	no GO terms	no GO terms
Eucgr.D00267	PF02362 - B3 DNA binding domain (B3) (1 of 98) OS=Egrandis	F:GO:0003677; C:GO:0005634	F:DNA binding; C:nucleus			IPR003340 (PFAM); IPR015300 (G3DSA:2.40.330.GENE3D); PTHR31920:SF26 (PANTHER); PTHR31920 (PANTHER); IPR003340 (PROSITE_PROFILES); IPR003340 (CDD); IPR015300 (SUPERFAMILY)	F:GO:0003677	F:DNA binding
Eucgr.D00268	PF02362 - B3 DNA binding domain (B3) (1 of 98) OS=Egrandis	F:GO:0003677; C:GO:0005634	F:DNA binding; C:nucleus			IPR015300 (G3DSA:2.40.330.GENE3D); IPR003340 (PFAM); PTHR31920 (PANTHER); PTHR31920:SF26 (PANTHER); IPR003340 (PROSITE_PROFILES); IPR003340 (CDD); IPR015300 (SUPERFAMILY)	F:GO:0003677	F:DNA binding
Eucgr.D00269	PF02362 - B3 DNA binding domain (B3) (1 of 98) OS=Egrandis	F:GO:0003677; C:GO:0005634	F:DNA binding; C:nucleus			IPR015300 (G3DSA:2.40.330.GENE3D); IPR003340 (PFAM); PTHR31920 (PANTHER); PTHR31920:SF26 (PANTHER); IPR003340 (PROSITE_PROFILES); IPR003340 (CDD); IPR015300 (SUPERFAMILY)	F:GO:0003677	F:DNA binding
Eucgr.D00270	PTHR12553//PTHR12553:SF47 - RIBONUCLEASE Z // SUBFAMILY NOT NAMED (1 of 1) OS=Egrandis					no IPS match	no IPS match	no IPS match
Eucgr.D00271	PTHR23172//PTHR23172:SF39 - AUXILIN/CYCLIN G-ASSOCIATED KINASE-RELATED // SUBFAMILY NOT NAMED (1 of 1) OS=Egrandis					mobidb-lite (MOBIDB_LITE); mobidb-lite (MOBIDB_LITE); mobidb-lite (MOBIDB_LITE); PTHR23172 (PANTHER); PTHR23172:SF39 (PANTHER)	no GO terms	no GO terms
Eucgr.D00272	PTHR24343:SF136 - CBL-INTERACTING SERINE/THREONINE-PROTEIN KINASE 23 (1 of 1) OS=Egrandis	F:GO:0004674; F:GO:0005524; P:GO:0006468; P:GO:0007165	F:protein serine/threonine kinase activity; F:ATP binding; P:protein phosphorylation; P:signal transduction	EC:2.7.11	Transferring phosphorus-containing groups	no IPS match	no IPS match	no IPS match
Eucgr.D00273	uncharacterized protein OS=Egrandis					mobidb-lite (MOBIDB_LITE); mobidb-lite (MOBIDB_LITE); mobidb-lite (MOBIDB_LITE); PTHR35101:SF9 (PANTHER); PTHR35101 (PANTHER)	no GO terms	no GO terms
Eucgr.D00275	uncharacterized protein OS=Egrandis					mobidb-lite (MOBIDB_LITE)	no GO terms	no GO terms
Eucgr.D00276	PTHR21495:SF40 - DIRIGENT PROTEIN 12-RELATED (1 of 12) OS=Egrandis	C:GO:0048046	C:apoplast			IPR004265 (PFAM); PTHR21495 (PANTHER); PTHR21495:SF40 (PANTHER)	no GO terms	no GO terms
Eucgr.D00277	PF01535//PF13041 - PPR repeat (PPR) // PPR repeat family (PPR_2) (1 of 235) OS=Egrandis	F:GO:0005515	F:protein binding			IPR002885 (PFAM); IPR011990 (G3DSA:1.25.40.GENE3D); IPR002885 (TIGRFAM); IPR002885 (PFAM); IPR011990 (G3DSA:1.25.40.GENE3D); IPR002885 (PFAM); IPR011990 (G3DSA:1.25.40.GENE3D); PTHR24015:SF863 (PANTHER); PTHR24015 (PANTHER); IPR002885 (PROSITE_PROFILES); IPR002885 (PROSITE_PROFILES); IPR002885 (PROSITE_PROFILES); IPR002885 (PROSITE_PROFILES); IPR002885 (PROSITE_PROFILES); IPR002885 (PROSITE_PROFILES); IPR002885 (PROSITE_PROFILES); IPR002885 (PROSITE_PROFILES); IPR002885 (PROSITE_PROFILES); IPR002885 (PROSITE_PROFILES); IPR002885 (PROSITE_PROFILES); IPR002885 (PROSITE_PROFILES); IPR002885 (PROSITE_PROFILES); IPR002885 (PROSITE_PROFILES); SSF81901 (SUPERFAMILY)	F:GO:0005515	F:protein binding
Eucgr.D00278	K10950 - ERO1-like protein alpha (ERO1L) (1 of 2) OS=Egrandis	F:GO:0003756; C:GO:0005783; C:GO:0016021; F:GO:0016671; P:GO:0055114	F:protein disulfide isomerase activity; C:endoplasmic reticulum; C:integral component of membrane; F:oxidoreductase activity, acting on a sulfur group of donors, disulfide as acceptor; P:oxidation-reduction process	EC:5.3.4.1	Protein disulfide-isomerase	no IPS match	no IPS match	no IPS match
Eucgr.D00281	K01251 - adenosylhomocysteinase (E3.3.1.1, ahcY) (1 of 6) OS=Egrandis	F:GO:0004013; P:GO:0006730; P:GO:0019510; F:GO:0051287	F:adenosylhomocysteinase activity; P:one-carbon metabolic process; P:S-adenosylhomocysteine catabolic process; F:NAD binding	EC:3.3.1.1	Adenosylhomocysteinase	IPR000043 (SMART); G3DSA:3.40.50.1480 (GENE3D); IPR000043 (PFAM); PTHR23420:SF12 (PANTHER); IPR000043 (PANTHER); SSF52283 (SUPERFAMILY)	no GO terms	no GO terms

Eucgr.D00282	K03538 - ribonuclease P protein subunit POP4 (POP4, RPP29) (1 of 1) OS=Egrandis	F:GO:0003723; F:GO:0004526; C:GO:0005730; P:GO:0008033; C:GO:0030677; P:GO:0090502	F:RNA binding; F:ribonuclease P activity; C:nucleolus; P:tRNA processing; C:ribonuclease P complex; P:RNA phosphodiester bond hydrolysis, endonucleolytic	EC:3.1.30; EC:3.1.26; EC:3.1.26.5	Acting on ester bonds; Acting on ester bonds; Ribonuclease P	no IPS match	no IPS match	no IPS match
Eucgr.D00284	PF12937 - F-box-like (F-box-like) (1 of 50) OS=Egrandis	F:GO:0005515	F:protein binding			IPR001810 (PFAM); G3DSA:1.20.1280.50 (GENE3D); mobidb-lite (MOBIDB_LITE); mobidb-lite (MOBIDB_LITE); mobidb-lite (MOBIDB_LITE); PTHR31215 (PANTHER); PTHR31215:SF2 (PANTHER); IPR001810 (PROSITE_PROFILES); IPR036047 (SUPERFAMILY)	F:GO:0005515	F:protein binding
Eucgr.D00285	uncharacterized protein OS=Egrandis					no IPS match	no IPS match	no IPS match
Eucgr.D00286	PF12937 - F-box-like (F-box-like) (1 of 50) OS=Egrandis	F:GO:0005515	F:protein binding			G3DSA:1.20.1280.50 (GENE3D); IPR001810 (PFAM); mobidb-lite (MOBIDB_LITE); mobidb-lite (MOBIDB_LITE); PTHR31215 (PANTHER); PTHR31215:SF2 (PANTHER); IPR001810 (PROSITE_PROFILES); IPR036047 (SUPERFAMILY)	F:GO:0005515	F:protein binding
Eucgr.D00287	2.6.1.13 - Ornithine aminotransferase / Ornithine--oxo-acid transaminase (1 of 1) OS=Egrandis	F:GO:0004587; F:GO:0030170; P:GO:0055129	F:ornithine-oxo-acid transaminase activity; F:pyridoxal phosphate binding; P:L-proline biosynthetic process	EC:2.6.1.13	Ornithine aminotransferase	no IPS match	no IPS match	no IPS match
Eucgr.D00288	K11517 - (S)-2-hydroxy-acid oxidase (HAO) (1 of 5) OS=Egrandis	F:GO:0010181; F:GO:0016491; P:GO:0055114	F:FMN binding; F:oxidoreductase activity; P:oxidation-reduction process			no IPS match	no IPS match	no IPS match
Eucgr.D00289	PTHR33077:SF17 - PROTEIN TIFY 5A-RELATED (1 of 2) OS=Egrandis					mobidb-lite (MOBIDB_LITE); mobidb-lite (MOBIDB_LITE)	no GO terms	no GO terms
Eucgr.D00290	uncharacterized protein OS=Egrandis					no IPS match	no IPS match	no IPS match
Eucgr.D00291	PTHR10543:SF42 - 9-CIS-EPOXYCAROTENOID DIOXYGENASE NCED2, CHLOROPLASTIC-RELATED (1 of 2) OS=Egrandis	F:GO:0016702; F:GO:0046872; P:GO:0055114	F:oxidoreductase activity, acting on single donors with incorporation of molecular oxygen, incorporation of two atoms of oxygen; F:metal ion binding; P:oxidation-reduction process	EC:1.13.11	Acting on single donors with incorporation of molecular oxygen (oxygenases). The oxygen incorporated need not be derived from O(2)	IPR004294 (PFAM); mobidb-lite (MOBIDB_LITE); mobidb-lite (MOBIDB_LITE); IPR004294 (PANTHER); PTHR10543:SF42 (PANTHER)	F:GO:0016702; P:GO:0055114	F:oxidoreductase activity, acting on single donors with incorporation of molecular oxygen, incorporation of two atoms of oxygen; P:oxidation-reduction process
Eucgr.D00292	2.7.7.49 - RNA-directed DNA polymerase / Revertase (1 of 28) OS=Egrandis					PTHR34676 (PANTHER); PTHR34676:SF2 (PANTHER)	no GO terms	no GO terms
Eucgr.D00293	PTHR32227:SF85 - GLYCOSYL HYDROLASES FAMILY PROTEIN (1 of 1) OS=Egrandis	F:GO:0004553; P:GO:0005975; C:GO:0016021	F:hydrolase activity, hydrolyzing O-glycosyl compounds; P:carbohydrate metabolic process; C:integral component of membrane			IPR000490 (PFAM); G3DSA:3.20.20.80 (GENE3D); PTHR32227:SF85 (PANTHER); PTHR32227 (PANTHER); IPR017853 (SUPERFAMILY)	F:GO:0004553; P:GO:0005975	F:hydrolase activity, hydrolyzing O-glycosyl compounds; P:carbohydrate metabolic process
Eucgr.D00295	K04507 - calcyclin binding protein (CACYBP, SIP) (1 of 1) OS=Egrandis	F:GO:0008171; P:GO:0032259; F:GO:0046983	F:O-methyltransferase activity; P:methylation; F:protein dimerization activity			mobidb-lite (MOBIDB_LITE)	no GO terms	no GO terms
Eucgr.D00296	PTHR23074//PTHR23074:SF92 - AAA ATPASE // SUBFAMILY NOT NAMED (1 of 1) OS=Egrandis	F:GO:0005524; C:GO:0005737; C:GO:0005874; F:GO:0008017; F:GO:0008568; F:GO:0016853; P:GO:0051013	F:ATP binding; C:cytoplasm; C:microtubule; F:microtubule binding; F:microtubule-severing ATPase activity; F:isomerase activity; P:microtubule severing	EC:3.6.1.3; EC:3.6.1.15; EC:3.6.4.3	Adenosinetriphosphatase; Nucleoside-triphosphate phosphatase; Microtubule-severing ATPase	no IPS match	no IPS match	no IPS match
Eucgr.D00297	PTHR10209:SF171 - GIBBERELLIN 2-BETA-DIOXYGENASE 2-RELATED (1 of 1) OS=Egrandis	F:GO:0016491; F:GO:0046872; P:GO:0055114	F:oxidoreductase activity; F:metal ion binding; P:oxidation-reduction process			IPR027443 (G3DSA:2.60.120.GENE3D); IPR026992 (PFAM); mobidb-lite (MOBIDB_LITE); PTHR10209 (PANTHER); PTHR10209:SF387 (PANTHER); SSF51197 (SUPERFAMILY)	no GO terms	no GO terms
Eucgr.D00298	PTHR11742:SF7 - ENDOPLASMIC RETICULUM MANNOSYL-OLIGOSACCHARIDE 1,2-ALPHA-MANNOSIDASE (1 of 1) OS=Egrandis	F:GO:0004571; F:GO:0005509; P:GO:0008152; C:GO:0016021	F:mannosyl-oligosaccharide 1,2-alpha-mannosidase activity; F:calcium ion binding; P:metabolic process; C:integral component of membrane	EC:3.2.1.113; EC:3.2.1.24	Mannosyl-oligosaccharide 1,2-alpha-mannosidase; Alpha-mannosidase	IPR001382 (PRINTS); IPR001382 (PFAM); IPR012341 (G3DSA:1.50.10.GENE3D); mobidb-lite (MOBIDB_LITE); mobidb-lite (MOBIDB_LITE); PTHR11742:SF55 (PANTHER); PTHR11742 (PANTHER); IPR036026 (SUPERFAMILY)	F:GO:0003824; F:GO:0004571; F:GO:0005509; C:GO:0016020	F:catalytic activity; F:mannosyl-oligosaccharide 1,2-alpha-mannosidase activity; F:calcium ion binding; C:membrane
Eucgr.D00299	PTHR22792:SF58 - LA-RELATED PROTEIN 6A (1 of 1) OS=Egrandis	F:GO:0003723; C:GO:0005634; P:GO:0006396; C:GO:1990904	F:RNA binding; C:nucleus; P:RNA processing; C:ribonucleoprotein complex			mobidb-lite (MOBIDB_LITE); mobidb-lite (MOBIDB_LITE); mobidb-lite (MOBIDB_LITE)	no GO terms	no GO terms
Eucgr.D00300	2.7.8.13 - Phospho-N-acetylmuramoyl-pentapeptide-transferase / UDP-MurNAc-pentapeptide phosphotransferase (1 of 1) OS=Egrandis	F:GO:0008963; C:GO:0016021	F:phospho-N-acetylmuramoyl-pentapeptide-transferase activity; C:integral component of membrane	EC:2.7.8.13	Phospho-N-acetylmuramoyl-pentapeptide-transferase	IPR000715 (PANTHER); PTHR22926:SF8 (PANTHER)	F:GO:0008963; C:GO:0016021	F:phospho-N-acetylmuramoyl-pentapeptide-transferase activity; C:integral component of membrane
Eucgr.D00301	K02955 - small subunit ribosomal protein S14e (RP-S14e, RPS14) (1 of 3) OS=Egrandis	F:GO:0003735; C:GO:0005840; P:GO:0006412	F:structural constituent of ribosome; C:ribosome; P:translation			no IPS match	no IPS match	no IPS match
Eucgr.D00302	K09832 - cytochrome P450, family 710, subfamily A (CYP710A) (1 of 1) OS=Egrandis	F:GO:0004497; F:GO:0005506; C:GO:0016021; F:GO:0016705; F:GO:0020037; P:GO:0055114	F:monooxygenase activity; F:iron ion binding; C:integral component of membrane; F:oxidoreductase activity, acting on paired donors, with incorporation or reduction of molecular oxygen; F:heme binding; P:oxidation-reduction process			IPR036396 (G3DSA:1.10.630.GENE3D); PTHR24286 (PANTHER); PTHR24286:SF0 (PANTHER); IPR036396 (SUPERFAMILY)	F:GO:0005506; F:GO:0016705; F:GO:0020037; P:GO:0055114	F:iron ion binding; F:oxidoreductase activity, acting on paired donors, with incorporation or reduction of molecular oxygen; F:heme binding; P:oxidation-reduction process
Eucgr.D00303	PTHR10052//PTHR10052:SF9 - 60S RIBOSOMAL PROTEIN L18A // SUBFAMILY NOT NAMED (1 of 6) OS=Egrandis	C:GO:0016021	C:integral component of membrane			mobidb-lite (MOBIDB_LITE)	no GO terms	no GO terms
Eucgr.D00304	K02882 - large subunit ribosomal protein L18Ae (RP-L18Ae, RPL18A) (1 of 2) OS=Egrandis	F:GO:0003735; C:GO:0005840; P:GO:0006412	F:structural constituent of ribosome; C:ribosome; P:translation			IPR023573 (PFAM); G3DSA:3.10.20.10 (GENE3D); IPR028877 (PANTHER); PTHR10052:SF19 (PANTHER); IPR028877 (HAMAP); SSF160374 (SUPERFAMILY)	F:GO:0003735; C:GO:0005840; P:GO:0006412	F:structural constituent of ribosome; C:ribosome; P:translation
Eucgr.D00305	PTHR11712:SF226 - 3-OXOACYL-[ACYL-CARRIER-PROTEIN] SYNTHASE I, CHLOROPLASTIC (1 of 1) OS=Egrandis	P:GO:0006633; F:GO:0016747	P:fatty acid biosynthetic process; F:transferase activity, transferring acyl groups other than amino-acyl groups			IPR014030 (PFAM); IPR016039 (G3DSA:3.40.47.GENE3D); PTHR11712:SF226 (PANTHER); PTHR11712 (PANTHER); IPR016039 (SUPERFAMILY)	F:GO:0003824	F:catalytic activity
Eucgr.D00306	PTHR13711:SF200 - HIGH MOBILITY GROUP B PROTEIN 14 (1 of 1) OS=Egrandis	F:GO:0003677; C:GO:0005634	F:DNA binding; C:nucleus			no IPS match	no IPS match	no IPS match
Eucgr.D00307	5.3.1.27 - 6-phospho-3-hexuloisomerase / Phospho-3-hexuloisomerase (1 of 2) OS=Egrandis	F:GO:0003824; C:GO:0016021	F:catalytic activity; C:integral component of membrane			no IPS match	no IPS match	no IPS match
Eucgr.D00308	uncharacterized protein OS=Egrandis					no IPS match	no IPS match	no IPS match
Eucgr.D00309	PTHR32468:SF17 - CATION/H(+) ANTIPORTER 10-RELATED (1 of 2) OS=Egrandis	F:GO:0015299; C:GO:0016021; P:GO:1902600	F:solute:proton antiporter activity; C:integral component of membrane; P:proton transmembrane transport			IPR006153 (PFAM); IPR038770 (G3DSA:1.20.1530.GENE3D); PTHR32468:SF17 (PANTHER); PTHR32468 (PANTHER)	P:GO:0006812; F:GO:0015299; C:GO:0016021; P:GO:0055085	P:cation transport; F:solute:proton antiporter activity; C:integral component of membrane; P:transmembrane transport
Eucgr.D00310	K00654 - serine palmitoyltransferase (SPT) (1 of 1) OS=Egrandis	F:GO:0005506; F:GO:0009055; P:GO:0009058; C:GO:0009535; C:GO:0009539; P:GO:0009767; C:GO:0016021; F:GO:0020037; F:GO:0030170	F:iron ion binding; F:electron transfer activity; P:biosynthetic process; C:chloroplast thylakoid membrane; C:photosystem II reaction center; P:photosynthetic electron transport chain; C:integral component of membrane; F:heme binding; F:pyridoxal phosphate binding			no IPS match	no IPS match	no IPS match
Eucgr.D00311	PTHR11945:SF229 - F23H11.23 PROTEIN-RELATED (1 of 4) OS=Egrandis	F:GO:0000977; C:GO:0005634; P:GO:0045944; F:GO:0046983	F:RNA polymerase II regulatory region sequence-specific DNA binding; C:nucleus; P:positive regulation of transcription by RNA polymerase II; F:protein dimerization activity			IPR002100 (PRINTS); IPR002100 (SMART); IPR002100 (PFAM); IPR036879 (G3DSA:3.40.1810.GENE3D); mobidb-lite (MOBIDB_LITE); PTHR11945 (PANTHER); PTHR11945:SF229 (PANTHER); IPR002100 (PROSITE_PROFILES); IPR033896 (CDD); IPR036879 (SUPERFAMILY)	F:GO:0000977; F:GO:0003677; P:GO:0045944; F:GO:0046983	F:RNA polymerase II regulatory region sequence-specific DNA binding; F:DNA binding; P:positive regulation of transcription by RNA polymerase II; F:protein dimerization activity
Eucgr.D00312	PTHR11945:SF229 - F23H11.23 PROTEIN-RELATED (1 of 4) OS=Egrandis	F:GO:0000977; C:GO:0005634; P:GO:0045944; F:GO:0046983	F:RNA polymerase II regulatory region sequence-specific DNA binding; C:nucleus; P:positive regulation of transcription by RNA polymerase II; F:protein dimerization activity			IPR002100 (PRINTS); IPR002100 (SMART); IPR002100 (PFAM); IPR036879 (G3DSA:3.40.1810.GENE3D); mobidb-lite (MOBIDB_LITE); PTHR11945 (PANTHER); PTHR11945:SF229 (PANTHER); IPR002100 (PROSITE_PROFILES); IPR033896 (CDD); IPR036879 (SUPERFAMILY)	F:GO:0000977; F:GO:0003677; P:GO:0045944; F:GO:0046983	F:RNA polymerase II regulatory region sequence-specific DNA binding; F:DNA binding; P:positive regulation of transcription by RNA polymerase II; F:protein dimerization activity
Eucgr.D00313	PTHR11945:SF229 - F23H11.23 PROTEIN-RELATED (1 of 4) OS=Egrandis	F:GO:0000977; C:GO:0005634; P:GO:0045944; F:GO:0046983	F:RNA polymerase II regulatory region sequence-specific DNA binding; C:nucleus; P:positive regulation of transcription by RNA polymerase II; F:protein dimerization activity			IPR002100 (PRINTS); IPR002100 (SMART); IPR036879 (G3DSA:3.40.1810.GENE3D); IPR002100 (PFAM); mobidb-lite (MOBIDB_LITE); mobidb-lite (MOBIDB_LITE); PTHR11945:SF229 (PANTHER); PTHR11945 (PANTHER); IPR002100 (PROSITE_PROFILES); IPR033896 (CDD); IPR036879 (SUPERFAMILY)	F:GO:0000977; F:GO:0003677; P:GO:0045944; F:GO:0046983	F:RNA polymerase II regulatory region sequence-specific DNA binding; F:DNA binding; P:positive regulation of transcription by RNA polymerase II; F:protein dimerization activity
Eucgr.D00314	K10735 - GINS complex subunit 4 (GINS4, SLD5) (1 of 1) OS=Egrandis	C:GO:0005634; P:GO:0006261	C:nucleus; P:DNA-dependent DNA replication			G3DSA:1.20.58.1030 (GENE3D); IPR036224 (SUPERFAMILY)	no GO terms	no GO terms

Eucgr.D00315	K09647 - mitochondrial inner membrane protease subunit 1 (IMP1) (1 of 3) OS=Egrandis	P:GO:0006508; F:GO:0008236; C:GO:0016021	P:proteolysis; F:serine-type peptidase activity; C:integral component of membrane			G3DSA:2.10.109.10 (GENE3D); IPR015927 (PFAM); PTHR12383 (PANTHER); PTHR12383:SF29 (PANTHER); cd06530 (CDD); IPR036286 (SUPERFAMILY)	no GO terms	no GO terms
Eucgr.D00316	PTHR30620//PTHR30620:SF30 - PERIPLASMIC BETA-GLUCOSIDASE-RELATED // SUBFAMILY NOT NAMED (1 of 2) OS=Egrandis	F:GO:0004553; P:GO:0005975	F:hydrolase activity, hydrolyzing O-glycosyl compounds; P:carbohydrate metabolic process			IPR026891 (SMART); IPR013783 (G3DSA:2.60.40.GENE3D); IPR026891 (PFAM); IPR036881 (G3DSA:3.40.50.GENE3D); PTHR42721 (PANTHER); PTHR42721:SF11 (PANTHER); IPR036881 (SUPERFAMILY)	F:GO:0004553; P:GO:0005975	F:hydrolase activity, hydrolyzing O-glycosyl compounds; P:carbohydrate metabolic process
Eucgr.D00317	PTHR12565:SF155 - TRANSCRIPTION FACTOR BHLH144 (1 of 1) OS=Egrandis	P:GO:0006355; F:GO:0046983	P:regulation of transcription, DNA-templated; F:protein dimerization activity			mobidb-lite (MOBIDB_LITE); mobidb-lite (MOBIDB_LITE); IPR024097 (PANTHER); PTHR12565:SF155 (PANTHER); IPR011598 (PROSITE_PROFILES); IPR036638 (SUPERFAMILY)	P:GO:0006355; F:GO:0046983	P:regulation of transcription, DNA-templated; F:protein dimerization activity
Eucgr.D00318	PTHR27000:SF16 - LRR RECEPTOR-LIKE SERINE/THREONINE-PROTEIN KINASE FLS2 (1 of 3) OS=Egrandis	F:GO:0004672; F:GO:0005515; F:GO:0005524; P:GO:0006468; C:GO:0016021	F:protein kinase activity; F:protein binding; F:ATP binding; P:protein phosphorylation; C:integral component of membrane			PR00019 (PRINTS); SM00365 (SMART); IPR003591 (SMART); IPR000719 (SMART); G3DSA:1.10.510.10 (GENE3D); IPR001611 (PFAM); IPR000719 (PFAM); IPR013210 (PFAM); G3DSA:3.30.200.20 (GENE3D); IPR032675 (G3DSA:3.80.10.GENE3D); IPR032675 (G3DSA:3.80.10.GENE3D); IPR032675 (G3DSA:3.80.10.GENE3D); IPR032675 (G3DSA:3.80.10.GENE3D); IPR001611 (PFAM); IPR032675 (G3DSA:3.80.10.GENE3D); PTHR43887 (PANTHER); IPR000719 (PROSITE_PROFILES); IPR011009 (SUPERFAMILY); SSF52047 (SUPERFAMILY); SSF52058 (SUPERFAMILY); SSF52058 (SUPERFAMILY)	F:GO:0004672; F:GO:0005515; F:GO:0005524; P:GO:0006468	F:protein kinase activity; F:protein binding; F:ATP binding; P:protein phosphorylation
Eucgr.D00319	K08912 - light-harvesting complex II chlorophyll a/b binding protein 1 (LHCB1) (1 of 5) OS=Egrandis	C:GO:0009522; C:GO:0009523; C:GO:0009535; P:GO:0009765; C:GO:0016021; F:GO:0016168; P:GO:0018298	C:photosystem I; C:photosystem II; C:chloroplast thylakoid membrane; P:photosynthesis, light harvesting; C:integral component of membrane; F:chlorophyll binding; P:protein-chromophore linkage			IPR023329 (G3DSA:1.10.3460.GENE3D); IPR022796 (PFAM); mobidb-lite (MOBIDB_LITE); IPR001344 (PANTHER); PTHR21649:SF55 (PANTHER); SSF103511 (SUPERFAMILY)	P:GO:0009765; C:GO:0016020	P:photosynthesis, light harvesting; C:membrane
Eucgr.D00320	K08912 - light-harvesting complex II chlorophyll a/b binding protein 1 (LHCB1) (1 of 5) OS=Egrandis	C:GO:0009522; C:GO:0009523; C:GO:0009535; P:GO:0009765; C:GO:0016021; F:GO:0016168; P:GO:0018298	C:photosystem I; C:photosystem II; C:chloroplast thylakoid membrane; P:photosynthesis, light harvesting; C:integral component of membrane; F:chlorophyll binding; P:protein-chromophore linkage			IPR023329 (G3DSA:1.10.3460.GENE3D); IPR022796 (PFAM); mobidb-lite (MOBIDB_LITE); PTHR21649:SF55 (PANTHER); IPR001344 (PANTHER); SSF103511 (SUPERFAMILY)	P:GO:0009765; C:GO:0016020	P:photosynthesis, light harvesting; C:membrane
Eucgr.D00321	K08912 - light-harvesting complex II chlorophyll a/b binding protein 1 (LHCB1) (1 of 5) OS=Egrandis	C:GO:0009522; C:GO:0009523; C:GO:0009535; P:GO:0009765; C:GO:0016021; F:GO:0016168; P:GO:0018298	C:photosystem I; C:photosystem II; C:chloroplast thylakoid membrane; P:photosynthesis, light harvesting; C:integral component of membrane; F:chlorophyll binding; P:protein-chromophore linkage			IPR023329 (G3DSA:1.10.3460.GENE3D); IPR022796 (PFAM); IPR001344 (PANTHER); PTHR21649:SF55 (PANTHER); SSF103511 (SUPERFAMILY)	P:GO:0009765; C:GO:0016020	P:photosynthesis, light harvesting; C:membrane
Eucgr.D00322	K08912 - light-harvesting complex II chlorophyll a/b binding protein 1 (LHCB1) (1 of 5) OS=Egrandis	C:GO:0009522; C:GO:0009523; C:GO:0009535; P:GO:0009765; C:GO:0016021; F:GO:0016168; P:GO:0018298	C:photosystem I; C:photosystem II; C:chloroplast thylakoid membrane; P:photosynthesis, light harvesting; C:integral component of membrane; F:chlorophyll binding; P:protein-chromophore linkage			IPR023329 (G3DSA:1.10.3460.GENE3D); IPR022796 (PFAM); mobidb-lite (MOBIDB_LITE); IPR001344 (PANTHER); PTHR21649:SF55 (PANTHER); SSF103511 (SUPERFAMILY)	P:GO:0009765; C:GO:0016020	P:photosynthesis, light harvesting; C:membrane
Eucgr.D00323	PTHR12526:SF304 - STARCH SYNTHASE 4, CHLOROPLASTIC/AMYLOPLASTIC-RELATED (1 of 1) OS=Egrandis	F:GO:0004373	F:glycogen (starch) synthase activity	EC:2.4.1.11	Glycogen(starch) synthase	no IPS match	no IPS match	no IPS match
Eucgr.D00324	PTHR31376:SF17 - PURINE PERMEASE 10-RELATED (1 of 8) OS=Egrandis	F:GO:0005215; C:GO:0016021	F:transporter activity; C:integral component of membrane			PF16913 (PFAM); PTHR31376:SF17 (PANTHER); IPR030182 (PANTHER); SSF103481 (SUPERFAMILY)	F:GO:0005215; C:GO:0016021	F:transporter activity; C:integral component of membrane
Eucgr.D00325	PTHR31376:SF17 - PURINE PERMEASE 10-RELATED (1 of 8) OS=Egrandis	F:GO:0005215; C:GO:0016021	F:transporter activity; C:integral component of membrane			PF16913 (PFAM); IPR030182 (PANTHER); PTHR31376:SF17 (PANTHER)	F:GO:0005215; C:GO:0016021	F:transporter activity; C:integral component of membrane
Eucgr.D00326	PTHR31376:SF17 - PURINE PERMEASE 10-RELATED (1 of 8) OS=Egrandis	F:GO:0005215; C:GO:0016021	F:transporter activity; C:integral component of membrane			PF16913 (PFAM); IPR030182 (PANTHER); PTHR31376:SF17 (PANTHER); SSF103481 (SUPERFAMILY)	F:GO:0005215; C:GO:0016021	F:transporter activity; C:integral component of membrane
Eucgr.D00327	PTHR31376:SF17 - PURINE PERMEASE 10-RELATED (1 of 8) OS=Egrandis	F:GO:0005215; C:GO:0016021	F:transporter activity; C:integral component of membrane			PF16913 (PFAM); mobidb-lite (MOBIDB_LITE); PTHR31376:SF17 (PANTHER); IPR030182 (PANTHER)	F:GO:0005215; C:GO:0016021	F:transporter activity; C:integral component of membrane
Eucgr.D00328	PTHR31376:SF17 - PURINE PERMEASE 10-RELATED (1 of 8) OS=Egrandis	F:GO:0005215; C:GO:0016021	F:transporter activity; C:integral component of membrane			PF16913 (PFAM); IPR030182 (PANTHER); PTHR31376:SF17 (PANTHER); SSF103481 (SUPERFAMILY)	F:GO:0005215; C:GO:0016021	F:transporter activity; C:integral component of membrane
Eucgr.D00330	PTHR31376:SF17 - PURINE PERMEASE 10-RELATED (1 of 8) OS=Egrandis	F:GO:0005215; C:GO:0016021	F:transporter activity; C:integral component of membrane			PF16913 (PFAM); IPR030182 (PANTHER); PTHR31376:SF17 (PANTHER)	F:GO:0005215; C:GO:0016021	F:transporter activity; C:integral component of membrane
Eucgr.D00331	PTHR31376:SF17 - PURINE PERMEASE 10-RELATED (1 of 8) OS=Egrandis	F:GO:0005215; C:GO:0016021	F:transporter activity; C:integral component of membrane			PF16913 (PFAM); IPR030182 (PANTHER); PTHR31376:SF17 (PANTHER); SSF103481 (SUPERFAMILY)	F:GO:0005215; C:GO:0016021	F:transporter activity; C:integral component of membrane
Eucgr.D00332	PTHR31376:SF17 - PURINE PERMEASE 10-RELATED (1 of 8) OS=Egrandis	F:GO:0005215; C:GO:0016021	F:transporter activity; C:integral component of membrane			PF16913 (PFAM); IPR030182 (PANTHER); PTHR31376:SF17 (PANTHER)	F:GO:0005215; C:GO:0016021	F:transporter activity; C:integral component of membrane
Eucgr.D00333	uncharacterized protein OS=Egrandis	C:GO:0016021	C:integral component of membrane			mobidb-lite (MOBIDB_LITE); mobidb-lite (MOBIDB_LITE); PTHR36715 (PANTHER)	no GO terms	no GO terms
Eucgr.D00334	uncharacterized protein OS=Egrandis	C:GO:0016021	C:integral component of membrane			no IPS match	no IPS match	no IPS match
Eucgr.D00335	2.3.1.45 - N-acetylneuraminate 7-O(or 9-O)-acetyltransferase (1 of 2) OS=Egrandis	C:GO:0016021	C:integral component of membrane			IPR012419 (PFAM); PTHR13533:SF22 (PANTHER); PTHR13533 (PANTHER)	no GO terms	no GO terms
Eucgr.D00336	PTHR31221:SF19 - WRKY TRANSCRIPTION FACTOR 28-RELATED (1 of 1) OS=Egrandis	F:GO:0003700; C:GO:0005634; P:GO:0006355; F:GO:0043565	F:DNA-binding transcription factor activity; C:nucleus; P:regulation of transcription, DNA-templated; F:sequence-specific DNA binding			IPR003657 (SMART); IPR003657 (PFAM); IPR036576 (G3DSA:2.20.25.GENE3D); PTHR31221 (PANTHER); PTHR31221:SF56 (PANTHER); IPR003657 (PROSITE_PROFILES); IPR036576 (SUPERFAMILY)	F:GO:0003700; P:GO:0006355; F:GO:0043565	F:DNA-binding transcription factor activity; P:regulation of transcription, DNA-templated; F:sequence-specific DNA binding
Eucgr.D00337	PTHR11003:SF126 - POTASSIUM INWARD RECTIFIER (KIR)-LIKE CHANNEL 3-RELATED (1 of 2) OS=Egrandis	F:GO:0005267; F:GO:0005509; C:GO:0016021; P:GO:0071805	F:potassium channel activity; F:calcium ion binding; C:integral component of membrane; P:potassium ion transmembrane transport			IPR003280 (PRINTS); IPR013099 (PFAM); G3DSA:1.10.287.70 (GENE3D); G3DSA:1.10.287.70 (GENE3D); mobidb-lite (MOBIDB_LITE); PTHR11003:SF126 (PANTHER); PTHR11003 (PANTHER); SSF81324 (SUPERFAMILY); SSF81324 (SUPERFAMILY)	F:GO:0005267; C:GO:0016020; P:GO:0071805	F:potassium channel activity; C:membrane; P:potassium ion transmembrane transport
Eucgr.D00338	K14801 - pre-rRNA-processing protein TSR4 (TSR4) (1 of 3) OS=Egrandis	C:GO:0005737; F:GO:0046872	C:cytoplasm; F:metal ion binding			mobidb-lite (MOBIDB_LITE); mobidb-lite (MOBIDB_LITE)	no GO terms	no GO terms
Eucgr.D00339	PTHR21535:SF49 - MAGNESIUM TRANSPORTER CORA-LIKE FAMILY PROTEIN (1 of 3) OS=Egrandis	C:GO:0016021; P:GO:0030001; F:GO:0046873; P:GO:0055085	C:integral component of membrane; P:metal ion transport; F:metal ion transmembrane transporter activity; P:transmembrane transport			mobidb-lite (MOBIDB_LITE); mobidb-lite (MOBIDB_LITE); mobidb-lite (MOBIDB_LITE); PTHR21535:SF50 (PANTHER); PTHR21535 (PANTHER)	no GO terms	no GO terms
